# 40 Hz light flickering alleviates chronic pain via adenosine signaling in the retina-amygdala pathway

**DOI:** 10.1038/s41422-026-01227-7

**Published:** 2026-03-04

**Authors:** Jiawang Chen, Tao Xu, Chenchen Zhang, Li Li, Yan He, Zhaoxia Sun, Jiasheng He, Zhimo Yao, Peng Cai, Yipeng Huang, Fenfen Ye, Wei Guo, Manli Jia, Jia Qu, Jiang-Fan Chen, Yi Zhang

**Affiliations:** 1 The Eye and Brain Center, State Key Laboratory of Eye Health, Eye Hospital, Wenzhou Medical University, Wenzhou, Zhejiang China; 2 Oujiang Laboratory, Zhejiang Lab for Regenerative Medicine, Vision and Brain Health, Wenzhou, Zhejiang China; 3 Zhejiang Provincial Key Laboratory of Medical Genetics, Key Laboratory of Laboratory Medicine, Ministry of Education, School of Laboratory Medicine and Life Sciences, Wenzhou Medical University, Wenzhou, Zhejiang China; 4 Institute of Genomic Medicine, Wenzhou Medical University, Wenzhou, Zhejiang China; 5 Key Laboratory of Pediatric Anesthesiology, Ministry of Education, Wenzhou Medical University, Wenzhou, Zhejiang China; 6 Key Laboratory of Precision Anesthesiology of Zhejiang Province, Department of Anesthesiology, the Second Affiliated Hospital and Yuying Children’s Hospital of Wenzhou Medical University, Wenzhou, Zhejiang China; 7 Wenzhou Municipal Key Laboratory of Neurodevelopmental Pathology and Physiology, the Second Affiliated Hospital and Yuying Children’s Hospital of Wenzhou Medical University, Wenzhou, Zhejiang China

**Keywords:** Molecular biology, Cell biology

## Abstract

Chronic pain affects over 20% of the global population, yet frontline treatments remain limited in efficacy and are often hampered by serious side effects. In search of novel and effective neuromodulation alternatives, we discovered that 40 Hz flickering light effectively alleviates inflammatory and neuropathic pain in mice. We identified the retina-central amygdala (CeA) pathway as a critical conduit for the analgesic effects of 40 Hz flickering light. Using circuit-specific manipulations, we demonstrated that activation of the retina-CeA pathway is both sufficient to mimic and necessary to mediate the analgesic outcomes of 40 Hz light stimulation. In terms of mechanism, we found that 40 Hz light flickering significantly increases extracellular adenosine levels in the CeA. Local pharmacological blockade of equilibrative nucleoside transporters prevented this adenosine increase and abolished the analgesic effects of 40 Hz light flickering, whereas focal adenosine infusion phenocopied the light-induced analgesia. Both interventions required A_2A_ receptor signaling to suppress nociceptive responses. Furthermore, we found that hyperalgesia could be destabilized in the CeA and reversed by 40 Hz light stimulation or adenosine infusion, mirroring memory reconsolidation processes and implicating the CeA as a key locus for pain memory erasure. Collectively, our findings demonstrate the multifaceted therapeutic benefits of 40 Hz light flickering as a novel non-invasive approach for pain management and reveal a distinct retina-CeA circuit and adenosine signaling mechanism for control of chronic pain and pain memory.

## Introduction

Chronic pain is a leading cause of disability and disease burden worldwide, affecting more than 20% of the population.^1,2^ Current treatments remain inadequate. Nonsteroidal anti-inflammatory drugs provide limited benefit for chronic pain and carry gastrointestinal, renal, and cardiovascular risks.^3,4^ Opioids, although effective for severe pain via µ-opioid receptor signaling,^5^ cause tolerance, dependence, and addiction, driving the global opioid crisis.^6^ These limitations highlight the urgent need for safe and effective pain therapies.

Chronic pain is sustained by maladaptive neuroplasticity in the central nervous system, including central sensitization and structural remodeling,^7,8^ which shape both sensory and affective aspects of pain.^9,10^ Sensitization extends from the spinal dorsal horn to higher centers, including the thalamus, central amygdala (CeA), anterior cingulate cortex, and somatosensory cortex.^11–14^ Long-term potentiation (LTP) and persistent changes in gene expression contribute to the maintenance of this sensitized state.^15^ In the spinal cord, LTP underlies injury-induced hyperalgesia and serves as a cellular model of pain memory.^15–17^ Pharmacological disruption of spinal LTP can reverse hyperalgesia.^18,19^ However, these systemic interventions are non-specific and unsafe. Targeting supraspinal circuits that encode pain memory may offer greater precision and durability. Among these, the CeA, a key hub for aversive learning and nociceptive integration,^20,21^ is a promising site for the regulation of persistent pain, but selective and safe modulation strategies remain unavailable.

Neuromodulation provides a non-invasive strategy for targeting dysfunctional brain circuits.^22,23^ Non-rhythmic light-based stimulation has shown analgesic effects in both patients and animal models, implicating specific eye-brain pathways, although the mechanisms remain unclear.^24–30^ We hypothesized that 40 Hz light flickering (gamma frequency) could serve as a novel non-invasive therapy for chronic pain. Adenosine signaling, particularly via A_1_ and A_2A_ receptors (A_1_R and A_2A_R), plays a critical role in pain modulation^31,32^ and metaplasticity control by raising thresholds for LTP induction.^33^ However, systemic adenosine delivery causes adverse cardiovascular and sedative effects.^34,35^ We therefore proposed that 40 Hz light flickering could trigger spatially targeted adenosine release through the retina-brain circuit to modulate pain pathways.

In this study, we systematically investigated the analgesic potential of light flickering across a range of frequencies and intensities in chronic pain models. Our findings revealed that light flickering elicited robust analgesic effects in a frequency- and intensity-dependent manner, with 40 Hz light stimulation yielding the most pronounced pain relief. Furthermore, 40 Hz light flickering exerted its analgesic effects by activating a direct retina-CeA pathway and by promoting local adenosine release within the CeA. Importantly, by locally engaging A_2A_R signaling to reshape dysfunctional pain circuits, this light stimulation not only attenuated ongoing hyperalgesia but also suppressed the formation and retrieval of pain memory. We therefore established a novel intervention to directly target a key pain-processing center, the CeA, via a dedicated visual pathway to induce naturally occurring adenosine. Taken together, these findings establish a non-invasive, retina-CeA-pathway-specific, and adenosine-mediated multifaceted approach for modulating central sensitization, offering a novel framework for developing targeted therapies for chronic pain.

## Results

### 40 Hz light flickering produces analgesia in chronic pain models

**Fig. 1:**
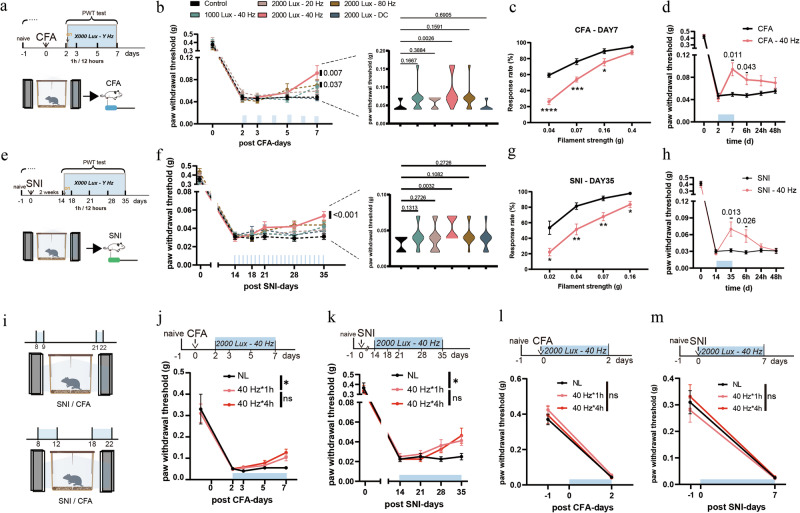
40 Hz light flickering alleviated mechanical hyperalgesia in chronic pain models. **a** Experimental design for light stimulation of CFA mice at different intensities and frequencies. PWT, paw withdrawal threshold. **b** PWT in CFA-model mice with or without 2-h daily light stimulation. Left: time course of PWT during light stimulation. Both 40 Hz and 80 Hz flickering light at 2000 lux significantly reduced mechanical hyperalgesia, as shown by increased PWT at CFA day 7 compared with the baseline (CFA day 2). *n* = 11 mice in each group. Right: between-group comparison of PWT at CFA day 7. CFA mice exposed to 40 Hz light at 2000 lux exhibited significant analgesia than in untreated controls. *n* = 11 mice in each group. **c** Paw withdrawal frequencies to von Frey filaments were significantly lower in CFA mice treated with 40 Hz, 2000 lux light compared with untreated controls. *n* = 13 mice in each group. **d** The analgesic effects of 40 Hz light at 2000 lux persisted for more than 6 h after cessation of stimulation in CFA mice. *n* = 16 mice in each group. **e** Experimental design for light stimulation in SNI mice at different intensities and frequencies. **f** PWTs in SNI mice with or without 2-h daily light stimulation. Left: time course of PWT. 40 Hz light flickering at 2000 lux significantly attenuated mechanical hyperalgesia, with the PWT at SNI day 35 elevated relative to the baseline (SNI day 14). *n* = 11 mice in each group. Right: between-group comparison of PWTs at SNI day 35, showing significant analgesia in the group treated with 40 Hz light at 2000 lux compared with untreated controls. *n* = 11 mice in each group. **g** Paw withdrawal frequencies to von Frey filaments were significantly lower in SNI mice treated with 40 Hz light at 2000 lux than in untreated controls. *n* = 14 mice in each group. **h** The analgesic effects of 40 Hz light at 2000 lux persisted for more than 6 h after cessation of stimulation in SNI mice. *n* = 12 mice in each group. **i** Experimental design for different durations of stimulation with 40 Hz light at 2000 lux. **j** PWTs in CFA mice under different durations of light stimulation: NL (no light stimulation), 40 Hz for 1 h (1 h per 12 h), and 40 Hz for 4 h (4 h per 12 h). Both treatment durations produced comparable analgesic effects. *n* = 8 mice in each group. **k** PWTs in SNI mice under the same light treatment regimens as in **j**, again showing similar analgesic efficacy between 1-h and 4-h stimulation. *n* = 8 mice in each group. **l** 40 Hz light flickering at 2000 lux did not affect the development of mechanical hyperalgesia in CFA mice. *n* = 8 mice in each group. **m** 40 Hz light flickering at 2000 lux did not affect the development of mechanical hyperalgesia in SNI mice. *n* = 8 mice in each group. Error bars represent SEM. Numerical labels indicate *P* values from within-group or between-group comparisons. For behavioral tests, *P < 0.05, **P < 0.01, ***P < 0.001, ****P < 0.0001; ns, not significant.

We investigated the antinociceptive effects of flickering light in two chronic pain models: the complete Freund’s adjuvant (CFA) model of inflammatory pain and the spared nerve injury (SNI) model of neuropathic pain (Fig. 1a, e). Unilateral intraplantar injection of CFA induces persistent inflammation and mechanical hyperalgesia.^36^ The SNI model, which mimics human neuropathic pain, reliably produces long-lasting allodynia and hyperalgesia.^37^

We first evaluated the analgesic efficacy of light stimulation at flickering frequencies of 20 Hz, 40 Hz, and 80 Hz, as well as direct current (DC) illumination, all delivered at 2000 lux in a chamber illuminated by LEDs. The treatment regimen consisted of two 1-h sessions per day: one in the morning and one 12 h later, totaling 2 h of stimulation daily. In the CFA model, 40 Hz stimulation at 2000 lux significantly alleviated mechanical hyperalgesia after 6 consecutive days of treatment relative to the baseline at CFA day 2 and untreated controls (Fig. 1b). On the basis of paw withdrawal frequency, such light stimulation significantly reduced mechanical hypersensitivity to non-noxious mechanical stimuli (0.07–0.16 g range) (Fig. 1c). The analgesic effects persisted for more than 6 h after stimulation ended, with partial but non-significant effects remaining thereafter (Fig. 1d). In SNI mice, the same 2-h daily regimen required 21 consecutive days to achieve significant analgesic effects (Fig. 1e–g). Similarly, the analgesia persisted for over 6 h after light cessation but diminished substantially thereafter (Fig. 1h). We next assessed thermal sensitivity following light stimulation. The 2000 lux 40 Hz flickering light did not significantly alter responses to either cold (4 °C) or heat (55 °C) stimuli in either model. Nonetheless, we observed a trend toward reduced cold and heat hypersensitivity in SNI mice, which did not reach statistical significance (Supplementary information, Fig. S1a).

Neither 20 Hz nor DC light produced significant analgesia (Fig. 1b, f). Although 80 Hz stimulation significantly reduced mechanical hyperalgesia in CFA mice relative to their baseline at CFA day 2 (*P* = 0.028), this effect was not statistically significant compared with untreated controls (*P* = 0.1591) (Fig. 1b), and only a mild, non-significant improvement was seen in SNI mice (Fig. 1f). Lowering the light intensity to 1000 lux at 40 Hz yielded only modest, non-significant improvements in both models (Fig. 1b, f). Collectively, these results showed that 40 Hz light flickering at 2000 lux produced the strongest and most reliable analgesic effects.

To determine whether extending daily exposure would enhance analgesia, we increased the stimulation to 4 h each in the morning and evening (8 h/day). However, the analgesic efficacy was comparable to that observed with the 2-h daily regimen in both models (Fig. 1i–k).

We also examined whether greater brightness would accelerate the therapeutic response. At 4000 lux, 40 Hz flickering for 2 h per day significantly shortened the treatment duration, alleviating hyperalgesia within 3 days in the CFA model and 7 days in the SNI model (Supplementary information, Fig. S1b–e). By contrast, 4000 lux DC light produced minimal effects during the same periods. Because prolonged exposure to high-intensity light (≥ 3000 lux) may cause stress, headaches, or retinal damage, subsequent experiments did not use such high-luminance light. In addition, in the formalin-induced spontaneous pain model, a single 1-h session of 40 Hz light flickering significantly reduced scratching behavior in Phase II compared with untreated controls, indicating a rapid, acute analgesic effect (Supplementary information, Fig. S1f, g). Finally, we explored the prophylactic potential of 40 Hz light flickering applied from the onset of injury. Initiating treatment on the day of CFA injection or SNI surgery failed to prevent the development of mechanical hyperalgesia (Fig. 1l, m). Once chronic pain was established, 2-h and 8-h daily regimens produced comparable analgesic effects. Therefore, a 2-h daily 40 Hz light stimulation at 2000 lux was used for all subsequent experiments.

### A 40 Hz light flickering-responsive analgesic ensemble in the CeA

**Fig. 2:**
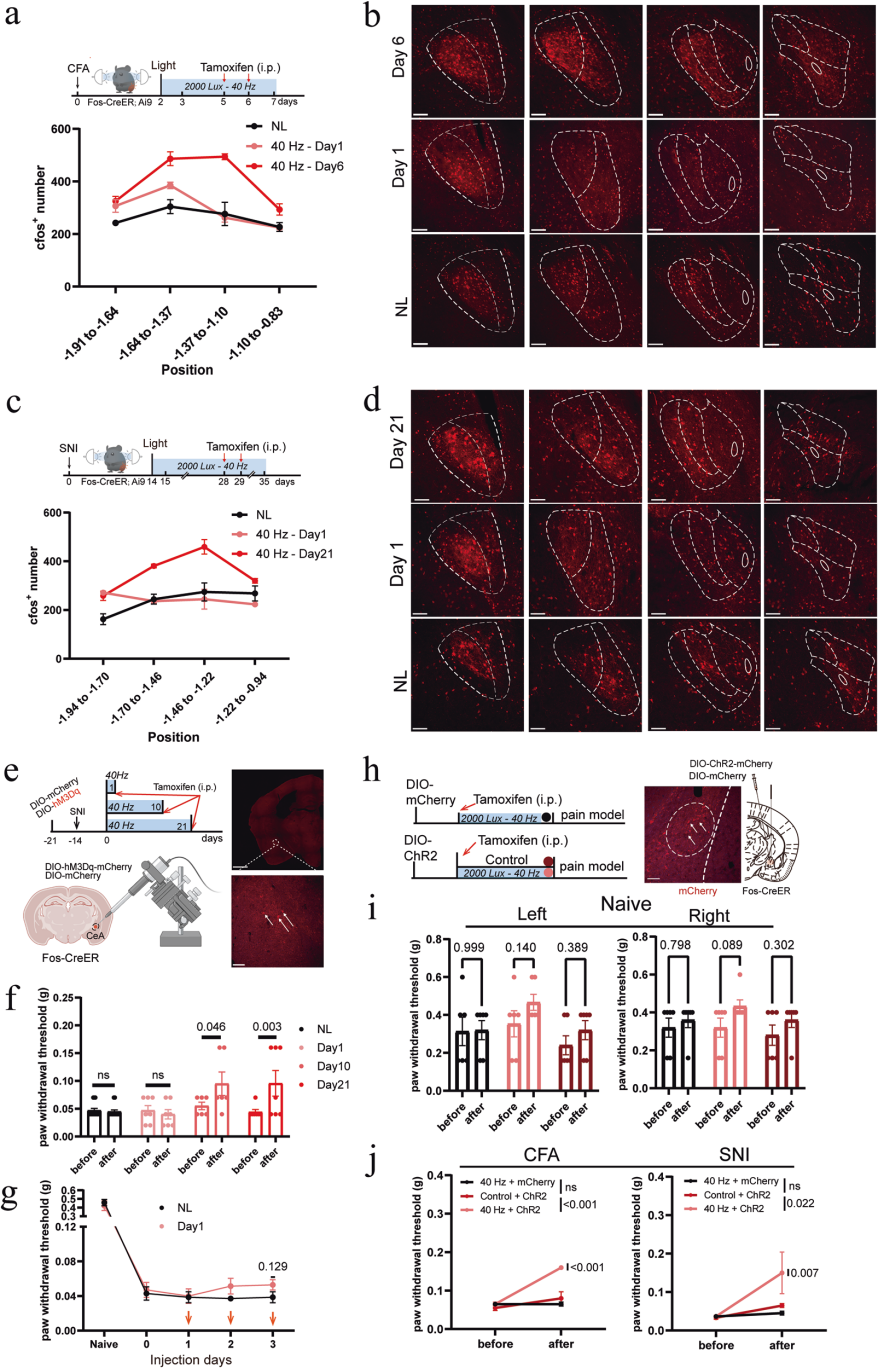
40 Hz light flickering activates analgesia-associated neurons in the CeA. **a** Top: TRAP2 mice were used to label CeA neurons activated by 40 Hz light flickering at 2000 lux in the CFA model. Bottom: counts of serial sections of TRAPed CeA neurons in CFA mice before and after 1 day or 6 days of light stimulation (*n* = 3 mice in each condition). **b** Representative serial sections showing TRAPed CeA neurons activated by 40 Hz light flickering at 2000 lux in CFA mice (Fos-CreERT2/TRAP2;Ai9). Scale bars, 100 μm. **c** Top: TRAP2 mice were used to label CeA neurons activated by 40 Hz light flickering at 2000 lux in the SNI model. Bottom: counts of serial sections of TRAPed CeA neurons in SNI mice before and after 1 day or 21 days of light stimulation (*n* = 3 mice in each condition). **d** Representative serial sections showing TRAPed CeA neurons activated by 40 Hz light flickering at 2000 lux in SNI mice (Fos-CreERT2/TRAP2;Ai9). Scale bars, 100 μm. **e** Left: Schematic of chemogenetic activation of TRAPed CeA neurons by 40 Hz light flickering at 2000 lux in SNI mice (Fos-CreERT2/TRAP2). Scale bars, 1 mm (top) and 100 μm (bottom). Right: Cre-dependent expression of hM3Dq in the CeA, indicated by arrows. **f** A single chemogenetic activation of TRAPed CeA neurons on either day 10 or day 21 significantly increased PWTs compared with the baseline in the SNI model. Error bars represent SEM. Numerical labels indicate *P* values from between-group comparisons. *n* = 6 or 7 mice in each group. **g** Repeatedly activating the TRAPed CeA neurons on day 1 for three consecutive days resulted in a modest increase in PWT. *n* = 6 or 7 mice in each group. **h** Left Schematic of optogenetic activation of TRAPed CeA neurons by 40 Hz light flickering at 2000 lux (Fos-CreERT2/TRAP2). Right: Cre-dependent expression of ChR2 in the CeA is indicated by arrows. Scale bar, 100 μm. **i** In naïve mice, optogenetic activation of TRAPed CeA neurons modestly increased the PWT compared with the baseline. Error bars represent SEM. Numerical labels indicate *P* values from between-group comparisons. *n* = 6 mice in each group. **j** In both the CFA and SNI models, optogenetic activation of TRAPed CeA neurons by 40 Hz light significantly increased the PWT compared with the baseline and controls. Error bars represent SEM. Numerical labels indicate *P* values from within-group or between-group comparisons. For behavioral tests, *n* = 6 mice in each condition. ns, not significant.

To identify neurons activated by 40 Hz light flickering, we used Fos-CreERT2/TRAP2;Ai9 mice, leveraging stimulus-transcription coupling via the immediate early gene *Fos*. This approach enables the genetic tagging of neurons activated during a defined time window. Accordingly, mice were first injected with CFA or underwent SNI surgery, followed by tamoxifen administration immediately after 40 Hz light treatment to tag the activated neuronal population. Animals were sacrificed one week later for imaging analysis (Fig. 2a–d). Quantification revealed robust activation in the CeA, as evidenced by significantly higher numbers of TRAPed neurons after 6 days (CFA) or 21 days (SNI) of 40 Hz stimulation compared with their respective pain-only controls. By contrast, 1 day of 40 Hz stimulation did not significantly increase the number of TRAPed CeA neurons in either model, with numbers comparable to those of untreated controls (Fig. 2a–d). TRAPed neurons in additional brain regions, including the visual cortex (V1) and insular cortex (IC), were also examined and quantified (Supplementary information, Fig. S2a–c). Representative whole-brain images from SNI mice are provided to illustrate the distribution of TRAPed neurons activated by 40 Hz light stimulation (Supplementary information, Fig. S2d).

To determine the functional contribution of this TRAPed ensemble, we performed chemogenetic activation in SNI mice (Fig. 2e). TRAP2 mice received intra-CeA injections of either AAV-Syn-DIO-hM3Dq or the control AAV-Syn-DIO-mCherry. One week later, mice underwent SNI surgery, and beginning on SNI day 14, they received 21 consecutive days of 40 Hz light stimulation before tamoxifen administration. Two weeks later, a single chemogenetic activation of the TRAPed CeA neurons with deschloroclozapine (DCZ, 1 mg/kg) in the absence of 40 Hz light significantly reduced mechanical hyperalgesia in these SNI mice (Fig. 2e, f). Thus, although some TRAPed cells may have been pain responsive, these findings indicate that the analgesic function of 40 Hz-activated CeA neurons dominated.

We next asked whether this ensemble remained stable across the 21-d stimulation period or shifted over time. To examine temporal dynamics, we TRAPed CeA neurons at early time points (day 1 or day 10 of stimulation) and later performed chemogenetic activation (Fig. 2e). Activation of CeA neurons TRAPed on day 10 produced robust analgesia in SNI mice with a single DCZ injection, whereas activation of neurons TRAPed on day 1 did not (Fig. 2f). Repeatedly activating the day-1 TRAPed population for three consecutive days elicited a delayed analgesic trend, although this effect did not reach statistical significance (Fig. 2g). These findings suggest that multiple stimulations are required to effectively capture the 40 Hz light-responsive analgesic ensemble in the CeA.

Finally, we asked whether TRAPed 40 Hz-responsive CeA neurons in naïve mice were sufficient to confer analgesic potential. TRAP2 mice were injected in the CeA with either AAV-Syn-DIO-mCherry or AAV-Syn-DIO-ChR2. After 3 weeks, mice were divided into three groups: (1) mCherry-injected mice exposed to 2 weeks of 40 Hz flickering (control), (2) ChR2-injected mice maintained under standard ambient light (control), and (3) ChR2-injected mice exposed to 2 weeks of 40 Hz flickering (experimental). Tamoxifen was administered immediately after the light treatment (Fig. 2h). Under naïve conditions, optogenetic activation of the TRAPed CeA neurons produced only a mild, non-significant increase in the paw withdrawal threshold (Fig. 2i). However, after inducing chronic pain with either SNI or CFA, optogenetic activation of these TRAPed CeA neurons by 40 Hz light significantly reduced mechanical hypersensitivity (Fig. 2j). Together, these results demonstrate that 40 Hz light flickering engages a distinct analgesic neuronal ensemble in the CeA. This ensemble forms progressively during repeated stimulation and is sufficient to confer potent analgesic effects across pain models, supporting the therapeutic potential of 40 Hz light flickering for chronic pain relief.

### The retina-CeA circuit is essential for analgesia induced by 40 Hz light flickering

**Fig. 3:**
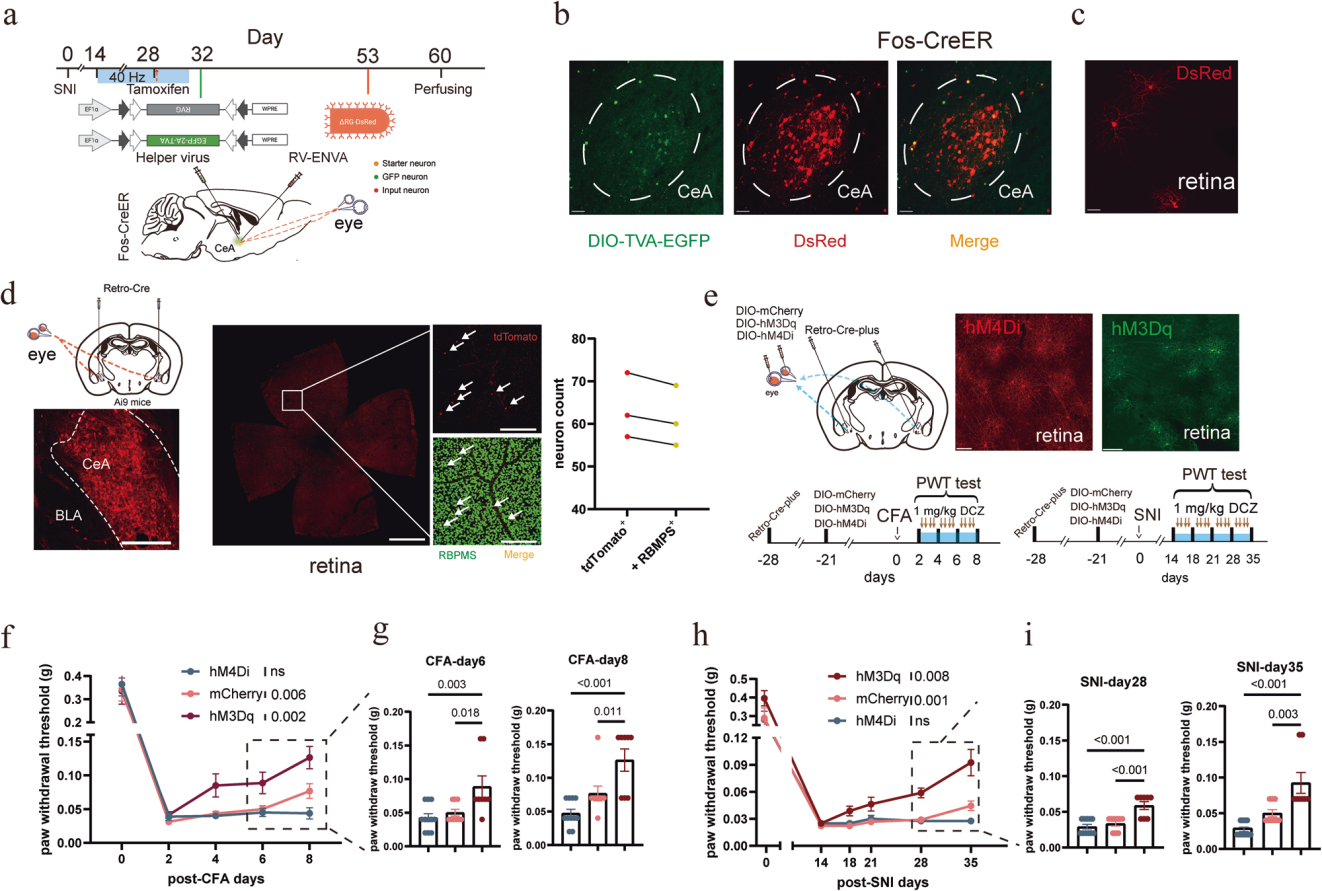
The retina-CeA pathway mediates analgesia induced by 40 Hz light flickering. **a** Schematic of RV-based monosynaptic tracing of TRAPed CeA neurons by 40 Hz light. **b** Representative images in the CeA showing the starter cells. Scale bars, 100 μm. **c** A representative retina image showing RV-DsRed-labeled RGCs. Scale bar, 100 μm. **d** Left: bilateral AAV2/Retro Plus-hSyn-Cre viral injections with a representative image of the injection site in the CeA. Scale bar, 100 μm. Middle: representative whole-mount retina image showing tdTomato-labeled RGCs co-labeled with the RGC marker RBPMS. Scale bars, 1 mm (100 μm for inset). Right: dot plot quantifying the co-localization of tdTomato and RBPMS signals. *n* = 3 mice in each group. **e** Left: schematic of chemogenetic manipulation targeting CeA-projecting RGCs. Right: Cre-dependent expression of inhibitory DREADD (hM4Di) and excitatory DREADD (hM3Dq) in the retina. Scale bars, 200 μm. **f** In the CFA model, chronic chemogenetic activation of CeA-projecting RGCs combined with 40 Hz light flickering significantly enhanced the analgesic effect compared with light stimulation alone. Chronic chemogenetic inhibition of CeA-projecting RGCs abolished 40 Hz light-induced analgesia. Error bars represent SEM. Numerical labels indicate *P* values from within-group comparisons: PWT at CFA day 8 vs the baseline at CFA day 2. For behavioral tests, *n* = 8 mice in the hM4Di group, *n* = 9 mice in the mCherry group, and *n* = 8 mice in the hM3Dq group. ns, not significant. **g** Summary of the results in **f**. Numerical labels indicate *P* values from between-group comparisons. **h** In the SNI model, chronic chemogenetic activation of CeA-projecting RGCs combined with 40 Hz light flickering similarly enhanced the analgesic effect of 40 Hz light flickering alone, whereas chronic chemogenetic inhibition abolished the light-induced analgesia. For behavioral tests, *n* = 8 mice in the hM4Di group, *n* = 9 mice in the mCherry group, and *n* = 8 mice in the hM3Dq group. ns, not significant. Error bars represent SEM. Numerical labels indicate *P* values from within-group comparisons: PWT at SNI day 35 vs the baseline at SNI day 14. **i** Summary of the results in **h**. Error bars represent SEM. Numerical labels indicate *P* values from between-group comparisons.

To investigate the neural circuitry underlying antinociception induced by 40 Hz light flickering, we used a rabies virus (RV)-based monosynaptic tracing strategy to map presynaptic inputs to CeA neurons activated by 40 Hz light flickering. In light-treated SNI mice, TRAPed CeA neurons were first infected with AAVs expressing the rabies glycoprotein and helper proteins (Fig. 3a). Twenty-one days later, we injected EnvA-pseudotyped SAD-∆G-DsRed RV into the CeA to selectively infect helper-expressing TRAPed CeA neurons (Fig. 3a). These double-labeled rabies-DsRed^+^/TVA-EGFP^+^ neurons served as the starter cells (Fig. 3b), which subsequently produced infectious ∆G-rabies-DsRed that propagated retrogradely to monosynaptically connected inputs. Excitingly and surprisingly, approximately a dozen retinal ganglion cells (RGCs) per retina were labeled (Fig. 3c).

To independently confirm the existence of a direct retina-CeA projection, we performed retrograde tracing by injecting AAV2/Retro Plus-hSyn-Cre into the CeA of Ai9 mice (Fig. 3d). This approach revealed robust labeling of RGCs, with approximately 60 fluorescently tagged cells per retina (Fig. 3d). Immunohistochemistry confirmed that over 95% of these were RBPMS-positive RGCs, supporting the existence of a direct retina-to-CeA pathway (Fig. 3d).

We performed additional retrograde tracing by injecting AAV2/Retro Plus-hSyn-Cre-EGFP into the CeA of wild-type (WT) mice (Supplementary information, Fig. S3a). Strong EGFP labeling was observed in several brain nuclei, including the anterior hypothalamic area (AHC), paraventricular thalamic nucleus (PVA), and insular cortex (GI, DI, and AIP), all of which are known regions that project to the CeA (Supplementary information, Fig. S3a). To assess the functional relevance of this retina-CeA circuit, we used chemogenetic tools to manipulate CeA-projecting RGCs. AAV2/Retro Plus-hSyn-Cre was bilaterally injected into the CeA, followed by intravitreal delivery of Cre-dependent AAVs encoding hM3Dq (activating DREADD), hM4Di (inhibitory DREADD), or mCherry (control) into the eyes (Fig. 3e). The expression of Cre-dependent hM3Dq and hM4Di in the retina was confirmed by the fluorescence signals of EGFP and mCherry, respectively (Fig. 3e). Chronic chemogenetic activation of CeA-projecting RGCs alone via intraperitoneal administration of DCZ (1 mg/kg, twice daily) for up to seven days tended to increase the paw withdrawal threshold in CFA mice compared with the control, but this effect did not reach statistical significance (Supplementary information, Fig. S3b, c). However, when combined with 40 Hz light flickering, chronic chemogenetic activation significantly enhanced analgesia in both CFA and SNI models compared with 40 Hz light flickering alone (Fig. 3f–i). More importantly, chronic chemogenetic activation combined with 40 Hz light flickering significantly shortened the duration required for light-induced analgesic effects in both pain models (Fig. 3f–i). These effects phenocopied those observed with increased light intensity. By contrast, chronic chemogenetic inhibition (DCZ, 1 mg/kg, twice daily right before light stimulation) of CeA-projecting RGCs abolished the analgesic effects of 40 Hz light stimulation (Fig. 3f–i).

**Fig. 4:**
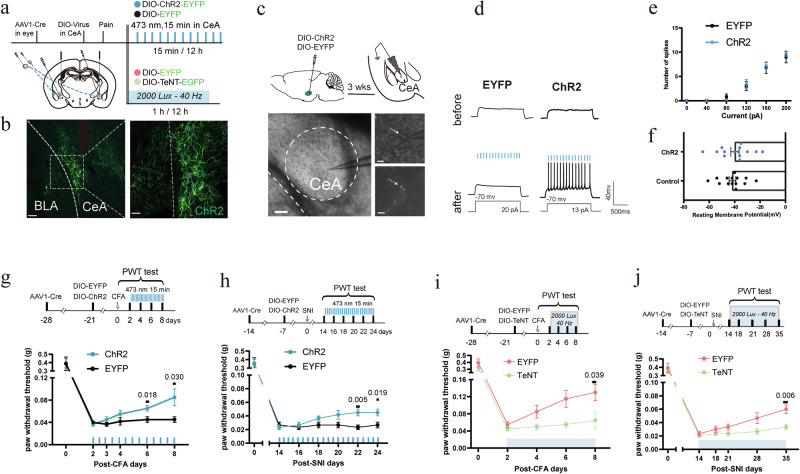
Retinorecipient CeA neurons mediate 40 Hz light flickering-induced analgesia. **a** Schematic of the experimental workflow. AAV1-hSyn-Cre was injected intravitreally to transduce RGCs, followed by intra-CeA injection of Cre-dependent AAVs expressing ChR2, TeNT, or EYFP. **b** Representative images showing ChR2 expression in CeA neurons receiving input from RGCs. Left: scale bars, 200 μm. Right: scale bar, 100 μm. **c** Top: schematic of the electrophysiological recording. AAV1-hSyn-Cre was injected intravitreally to transduce RGCs, followed by intra-CeA injection of Cre-dependent AAVs expressing ChR2 or EYFP. Bottom: representative bright-field (left) and fluorescent (right) images of acute amygdala slices. Left: scale bar, 200 μm. Right: scale bars, 50 μm. **d** Representative traces of whole-cell current-clamp recordings obtained from CeA neurons transduced with EYFP (left) or ChR2 (right) and irradiated with a 473-nm laser. Action potentials were elicited using a 3-s depolarizing current injection that evoked a train of action potentials (APs) after the laser application in the ChR2 group. In the EYFP group, no APs were elicited after the laser irradiation. Injected current amplitudes are illustrated under the voltage records. **e** Evoked spikes by currents were analyzed for CeA neurons expressing EYFP and ChR2. Values are presented as mean ± SEM. **f** Resting membrane potentials were analyzed for CeA neurons expressing EYFP and ChR2. Values are presented as mean ± SEM. **g** Chronic optogenetic activation of retinorecipient CeA neurons significantly increased the PWT in CFA mice compared with the control. Error bars represent SEM. Numerical labels indicate *P* values from between-group comparisons. For behavioral tests, *n* = 6 mice in each group. **h** Chronic optogenetic activation of retinorecipient CeA neurons significantly increased the PWT in SNI mice compared with the control. Error bars represent SEM. Numerical labels indicate *P* values from between-group comparisons. For behavioral tests, *n* = 6 mice in each group. **i** Silencing the retina-CeA pathway via TeNT expression abolished the analgesic effect of 40 Hz light flickering in CFA mice. Error bars represent SEM. Numerical labels indicate *P* values from between-group comparisons. For behavioral tests, *n* = 6 mice in each group. **j** Silencing the retina-CeA pathway via TeNT expression abolished the analgesic effect of 40 Hz light flickering in SNI mice. Error bars represent SEM. Numerical labels indicate *P* values from between-group comparisons. For behavioral tests, *n* = 6 mice in each group.

To further confirm this circuit, we directly stimulated the retinorecipient CeA neurons using an anterograde labeling strategy (Fig. 4a). One week after bilateral intravitreal injection of the anterograde spreading AAV1-Cre, we injected AAV-hSyn-DIO-ChR2 or AAV-hSyn-DIO-EYFP (control) into the CeA (Fig. 4b). Acute brain slice recordings demonstrated that blue light stimulation in ChR2-expressing retinorecipient CeA neurons reliably evoked trains of action potentials (Fig. 4c, d). Furthermore, our results confirmed that neither ChR2 nor EYFP expression altered the electrophysiological properties of the recorded CeA neurons (Fig. 4e, f). Four weeks after viral injections, mice were subjected to either a CFA injection or SNI surgery. Subsequently, the injured mice underwent twice-daily 15-min optogenetic stimulation sessions. This activation significantly reduced mechanical hyperalgesia after five consecutive days of stimulation in CFA mice and nine days in SNI mice (Fig. 4g, h), demonstrating the sufficiency of the pathway in mediating analgesia. Next, we tested the necessity of the retina-CeA circuit by expressing tetanus neurotoxin (TeNT) in the retinorecipient CeA neurons. Following bilateral intravitreal injection of AAV1-Cre, mice received CeA injections of either AAV-hSyn-DIO-TeNT or AAV-hSyn-DIO-EYFP (control). Blocking the synaptic output of retinorecipient CeA neurons abolished the analgesic effects of 40 Hz light flickering in both pain models (Fig. 4i, j), demonstrating that intact synaptic transmission in this circuit is necessary for mediating antinociception.

**Fig. 5:**
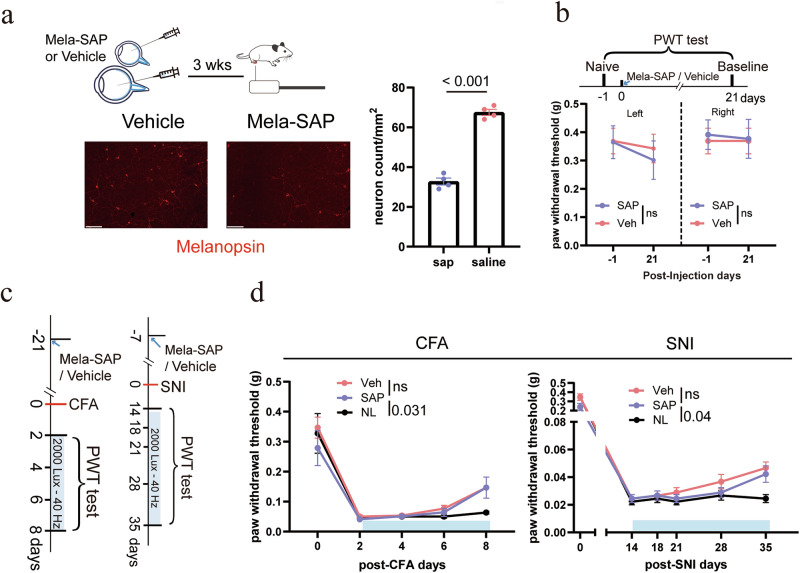
Melanopsin-expressing RGCs are not required for 40 Hz light flickering-induced analgesia. **a** Top: experimental design. Intravitreal injection of melanopsin-saporin (Mela-SAP) was used to ablate ipRGCs, followed by behavioral testing using the von Frey assay. Bottom: representative retinal whole-mount images showing melanopsin-positive cells (UF008 antibody) after Mela-SAP injection, confirming targeted ablation. Scale bars, 100 μm. For cell counting, *n* = 3 mice in each group, *P* < 0.001. **b** Mela-SAP-mediated ablation of ipRGCs did not alter the PWT under naïve conditions. Error bars represent SEM. Numerical labels indicate *P* values from between-group comparisons. For behavioral tests, *n* = 11 mice in each group. ns, not significant. **c** Experimental design for Mela-SAP ablation in the CFA and SNI models. **d** Mela-SAP ablation of ipRGCs did not impair 40 Hz light flickering-induced analgesia in CFA or SNI model mice. Error bars represent SEM. Numerical labels indicate *P* values from between-group comparisons. For behavioral tests, *n* = 9 mice in each group. ns, not significant.

Finally, we examined the role of intrinsically photosensitive RGCs (ipRGCs), which are known to mediate non-image-forming light responses. To selectively ablate ipRGCs, melanopsin-conjugated saporin (melanopsin-SAP) was bilaterally injected into the vitreous humor (Fig. 5a). Melanopsin-SAP effectively ablated ipRGCs by 70% (Fig. 5a). Three weeks post-injection, melanopsin-SAP-treated mice displayed no significant change in baseline paw withdrawal threshold (Fig. 5b). Importantly, the antinociceptive effects of 40 Hz light flickering remained intact in both CFA and SNI models, despite ablation of ipRGCs (Fig. 5c, d). These results indicate that ipRGCs are not essential for the analgesia induced by 40 Hz light flickering.

Together, these data define a novel retina-CeA visual pathway that is both necessary and sufficient for the analgesia induced by 40 Hz flickering, acting independently of ipRGCs and relying on conventional RGCs to elicit pain relief.

### Adenosine signaling in the CeA mediates the analgesia induced by 40 Hz light flickering

**Fig. 6:**
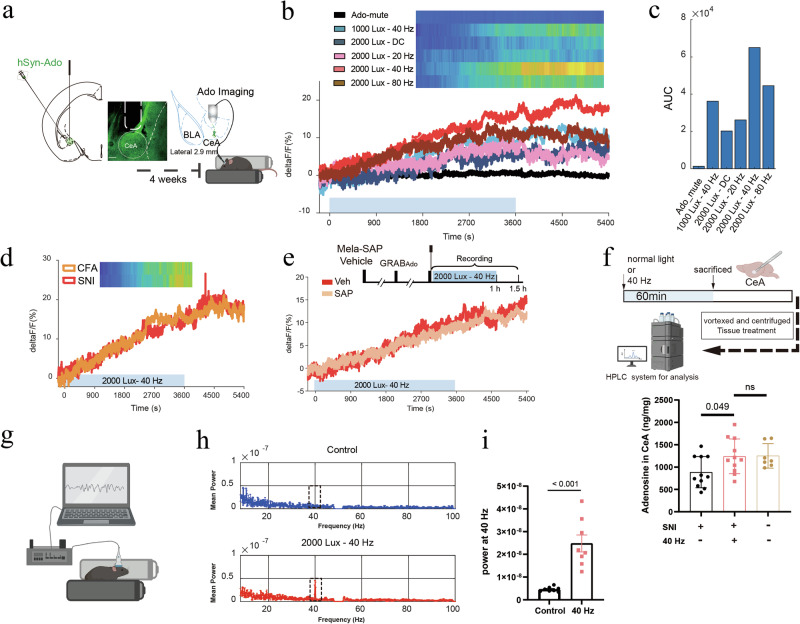
40 Hz light flickering increases extracellular adenosine levels in a light frequency- and intensity-dependent manner in the CeA in correlation with LFP gamma power. **a** Experimental design for adenosine recording during light stimulation. Middle: a representative image showing expression of the adenosine sensor in the CeA. Scale bar, 100 μm. **b** Effects of light flickering at different intensities and frequencies on extracellular adenosine levels in the CeA. *n* = 6 mice in each group. **c** Quantification of light flickering-evoked adenosine signals in the CeA, with AUC values showing that 40 Hz light flickering at 2000 lux yielded the greatest increase. *n* = 6 mice in each group. **d** 40 Hz light flickering at 2000 lux elicited a similar increase in extracellular adenosine levels in the CeA in both CFA and SNI models. *n* = 6 mice in each group. **e** Ablation of ipRGCs by Mela-SAP did not affect the light flickering-induced increase in extracellular adenosine levels in the CeA. *n* = 6 mice in each group. **f** Top: HPLC assays were used to quantify total adenosine levels in the CeA. Bottom: total adenosine levels in the CeA were significantly lower in SNI mice compared with naïve controls but were restored by 40 Hz light flickering at 2000 lux. **g** Workflow for multichannel LFP recording in the CeA during light treatment. **h** Spectral power density of LFP in the CeA for control vs light treatment groups. The area enclosed by the dashed line indicates the position of 40 Hz ± 2 Hz. *n* = 8 mice in each group. **i** 40 Hz flickering at 2000 lux significantly increased gamma-band power in the CeA under light stimulation compared with the control (*P* < 0.001). Error bars represent SEM. *n* = 8 mice in each group.

Previous studies have suggested a role for adenosine in the changes in brain function induced by 40 Hz light flickering.^38,39^ To investigate whether adenosine might be involved in the observed analgesic effects, we used a highly sensitive and selective adenosine sensor (GRABAdo) to monitor extracellular adenosine dynamics in the CeA during light stimulation.^40^ Following the injection of an AAV expressing GRABAdo or a non-binding mutant of GRABAdo (GRABAdo-mut) into the CeA, as depicted in Fig. 6a, we used fiber photometry to measure fluorescence signals that reflected extracellular adenosine levels.

Upon exposure to 40 Hz light flickering at 2000 lux, a robust and sustained increase in extracellular adenosine concentrations was observed in the CeA, with fluorescence intensity (ΔF/F) remaining elevated, up to 20% above baseline, for at least 30 min post-stimulation (Fig. 6b). We next examined the effect of different light frequencies and intensities on extracellular adenosine production in the CeA. Whereas 40 Hz light flickering at 1000 lux and 80 Hz light flickering at 2000 lux elicited moderate increases in extracellular adenosine levels in the CeA, 20 Hz light flickering and DC at 2000 lux produced marginal changes (Fig. 6b, c). These results demonstrate that adenosine release is dependent on both the frequency and intensity of light. In both the CFA and SNI models, 40 Hz light flickering at 2000 lux produced similar significant increases in extracellular adenosine levels in the CeA, comparable to those observed in naïve mice (Fig. 6d). Importantly, the increase in extracellular adenosine in the CeA was unaffected by melanopsin-SAP treatment (Fig. 6e).

To confirm these findings, we performed HPLC assays to measure the increase in endogenous adenosine levels in the CeA induced by 40 Hz light flickering. In SNI mice, total adenosine levels in the CeA were markedly reduced compared with those of naïve controls. Notably, 1 h of 40 Hz light stimulation significantly increased total adenosine levels in the CeA of SNI mice, restoring them to levels comparable to those observed in naïve mice (Fig. 6f). Moreover, electrophysiological recordings of local field potentials revealed that 40 Hz light flickering at 2000 lux entrained gamma-band oscillations in the CeA, directly coupling rhythmic visual input with local neural activity (Fig. 6g–i).

**Fig. 7:**
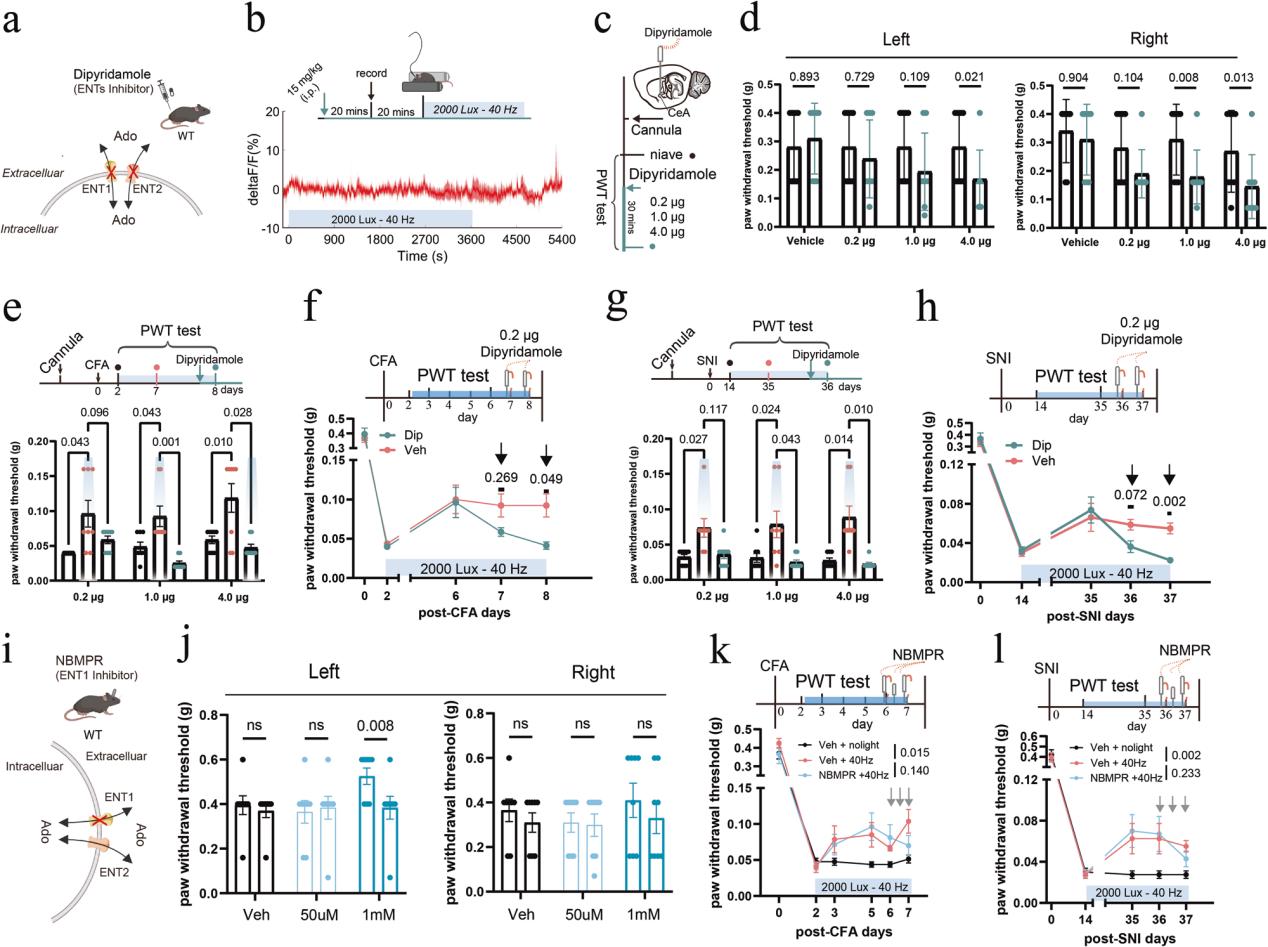
Dipyridamole antagonizes 40 Hz light flickering-induced analgesia. **a** Schematic illustration of the release of adenosine via ENTs and pharmacological inhibition of ENTs by dipyridamole administration. **b** Top: experimental design for in vivo adenosine measurement following intraperitoneal dipyridamole administration. Bottom: intraperitoneal injection of dipyridamole abolished the light flickering-induced increase in extracellular adenosine in the CeA. *n* = 6 mice. **c** Schematic showing cannula implantation in the CeA for local dipyridamole delivery and von Frey testing in naïve mice. **d** In naïve mice, intra-CeA dipyridamole injection induced a dose-dependent decrease in the PWT. Error bars represent SEM. Numerical labels indicate *P* values from between-group comparisons. For behavioral tests, *n* = 8 mice in each group. **e** Intra-CeA administration of dipyridamole at a dose of 1.0 μg or 4.0 μg effectively reversed the analgesic effects of light flickering in CFA mice. Error bars represent SEM. Numerical labels indicate *P* values from between-group comparisons. For behavioral tests, *n* = 8 mice in each group. **f** Repeated administration of a low dose (0.2 μg) of intra-CeA dipyridamole over 2 d also effectively reversed light flickering-induced analgesia in CFA mice, suggesting cumulative effects. Error bars represent SEM. Numerical labels indicate *P* values from between-group comparisons. For behavioral tests, *n* = 8 mice in each group. **g** Intra-CeA dipyridamole at a dose of 1.0 μg or 4.0 μg similarly reversed light flickering-induced analgesia in SNI mice. Error bars represent SEM. Numerical labels indicate *P* values from between-group comparisons. For behavioral tests, *n* = 8 mice in each group. **h** Repeated low doses (0.2 μg) of intra-CeA dipyridamole over 2 d effectively blocked the analgesic effects of light flickering in SNI mice, again indicating cumulative action. Error bars represent SEM. Numerical labels indicate *P* values from between-group comparisons. For behavioral tests, *n* = 8 mice in each group. **i** Schematic illustrating adenosine release via ENTs and pharmacological inhibition of ENT1 through intra-CeA administration of NBMPR. **j** In naïve mice, intra-CeA NBMPR administration produced a dose-dependent reduction in the PWT. Error bars represent SEM. Numerical labels indicate *P* values from between-group comparisons. For behavioral tests, *n* = 8 mice in each group. **k** In CFA mice, three consecutive daily intra-CeA injections of a low dose of NBMPR (2 μL of 50 μM) showed a tendency to partially attenuate the analgesic effects induced by 40 Hz light flickering. Error bars represent SEM. Numerical labels indicate *P* values from between-group comparisons. For behavioral tests, *n* = 8 mice in each group. **l** In SNI mice, three consecutive daily intra-CeA injections of a low dose of NBMPR (2 μL of 50 μM) showed a tendency to partially attenuate 40 Hz light flickering-induced analgesia. Error bars represent SEM. Numerical labels indicate *P* values from between-group comparisons. For behavioral tests, *n* = 8 mice in each group.

Because extracellular adenosine primarily arises from equilibrative nucleoside transporter (ENT)-mediated efflux of intracellular adenosine generated during ATP consumption,^41,42^ we next assessed the contribution of ENT1/2-dependent transmembrane transport to 40 Hz light-induced adenosine elevation in the CeA. Pretreatment with dipyridamole (15 mg/kg, i.p.),^43^ an ENT1/2 inhibitor administered 40 min before the light-flickering stimulus, completely abolished any increase in extracellular adenosine in response to 40 Hz light (Fig. 7a, b). We also examined the effects of direct dipyridamole administration in the right CeA on mouse pain behaviors (Fig. 7c). The results showed that various doses of dipyridamole negatively affected the paw withdrawal threshold of naïve mice (Fig. 7d). Administration of dipyridamole at higher doses of 1.0 μg or 4.0 μg effectively abolished the analgesic effects induced by 40 Hz light flickering in both CFA and SNI models (Fig. 7e, g). Even a lower dose of 0.2 μg dipyridamole, administered continuously over two days, significantly counteracted the analgesic effects of 40 Hz light flickering (Fig. 7f, h).

Because dipyridamole has multiple pharmacological targets in addition to ENT1/2 inhibition,^44^ we tested the contribution of ENT1 using the highly selective inhibitor NBMPR, which is the gold-standard antagonist for ENT1^45^ (Fig. 7i). Prior studies have shown that microinjection of 50 μM NBMPR into the CeA at a dose of 0.5 μL reduced anxiety-like behavior,^46^ indicating the functional role of ENT1 in the CeA. Here, we evaluated two doses of NBMPR delivered to the right CeA of naïve mice (2 μL of 50 μM or 2 μL of 1 mM). Whereas the higher dose (2 μL of 1 mM) altered baseline behaviors, the lower dose (2 μL of 50 μM) did not affect mechanical sensitivity in naïve animals (Fig. 7j) and was therefore selected for subsequent experiments. In both CFA and SNI models, NBMPR was microinjected into the CeA 15 min before each 40 Hz light stimulation. Three consecutive NBMPR administrations showed a tendency to partially attenuate the analgesic effects of 40 Hz light flickering; however, this effect did not reach statistical significance in either model (Fig. 7k, l). Given that 40 Hz light flickering robustly increases intracellular adenosine production, ENTs are expected to function predominantly in the efflux mode under these conditions. These results are supported by our previous observations in the V1 using ENT knockout mice, in which ENT2 knockout nearly abolished the 40 Hz light-induced increase in extracellular adenosine, whereas ENT1 knockout only partially attenuated this response.^38^ The preferential involvement of ENT2 may be attributable to its higher transport capacity and approximately fourfold higher expression in neurons and astrocytes, which would enable more efficient elevation of extracellular adenosine during light flickering.^47,48^ Together, these findings suggest that both ENT1 and ENT2 may contribute to the 40 Hz light-induced elevation of extracellular adenosine in the CeA, with ENT2 likely playing a more prominent role.

**Fig. 8:**
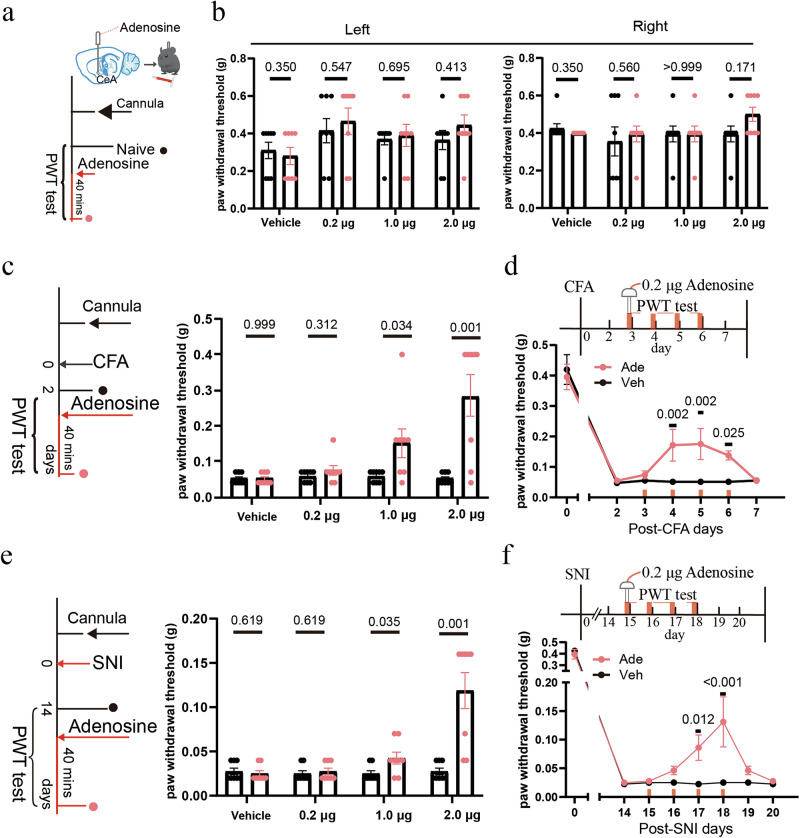
Intra-CeA adenosine administration alleviates mechanical hypersensitivity in pain models. **a** Schematic of intra-CeA adenosine administration in naïve mice. **b** Compared with the baseline, no significant changes were observed in the PWT of naïve mice following intra-CeA adenosine administration at various doses (vehicle, 0.2 μg, 1.0 μg, and 2.0 μg). Error bars represent SEM. Numerical labels indicate *P* values from between-group comparisons. For behavioral tests, *n* = 8 mice in each group. **c** Left: schematic of intra-CeA adenosine delivery in CFA mice. Right: intra-CeA administration of adenosine at doses of 1.0 μg and 2.0 μg significantly increased the PWT in the CFA model. Error bars represent SEM. Numerical labels indicate *P* values from between-group comparisons. For behavioral tests, *n* = 8 mice in each group. **d** Repeated intra-CeA administration of a low dose (0.2 μg) of adenosine over 2 d progressively increased the PWT of CFA mice, indicating cumulative analgesic effects. Error bars represent SEM. Numerical labels indicate *P* values from between-group comparisons. For behavioral tests, *n* = 8 mice in each group. **e** Left: schematic of intra-CeA adenosine delivery in SNI mice. Right: intra-CeA administration of adenosine at doses of 1.0 μg and 2.0 μg significantly increased the PWT in the SNI model. Error bars represent SEM. Numerical labels indicate *P* values from between-group comparisons. For behavioral tests, *n* = 8 mice in each group. **f** Repeated intra-CeA administration of a low dose (0.2 μg) of adenosine over 3 d also produced cumulative analgesic effects in SNI mice, as evidenced by increased PWT. Error bars represent SEM. Numerical labels indicate *P* values from between-group comparisons. For behavioral tests, *n* = 8 mice in each group.

To determine whether direct adenosine administration could mimic 40 Hz light-induced analgesia, we infused various doses of adenosine (0.2 μg, 1.0 μg, and 2.0 μg) into the right CeA. In naïve mice, none of these adenosine doses significantly affected paw withdrawal thresholds (Fig. 8a, b). We then applied the same doses of adenosine via cannula to the right CeA of CFA and SNI mice. Notably, higher doses of adenosine (1.0 μg and 2.0 μg) rapidly alleviated mechanical hyperalgesia in these models (Fig. 8c, e). A lower dose of adenosine at 0.2 μg did not immediately reduce mechanical hypersensitivity. However, following > 2 days of consecutive administration, 0.2 μg adenosine also significantly increased the paw withdrawal threshold in CFA and SNI mice (Fig. 8d, f). In our HPLC analysis, the total adenosine content of the CeA differed by 0.13 µg between naïve and SNI mice, and 40 Hz light stimulation increased adenosine levels in SNI mice by 0.11 µg, an amount comparable to the lower dose (0.2 µg) used in our in vivo pharmacological assays. Consistent with these measurements, our pharmacological experiments demonstrated that increasing extracellular adenosine in the CeA is sufficient to produce antinociceptive effects and that these effects are cumulatively enhanced by repeated daily administration.

**Fig. 9:**
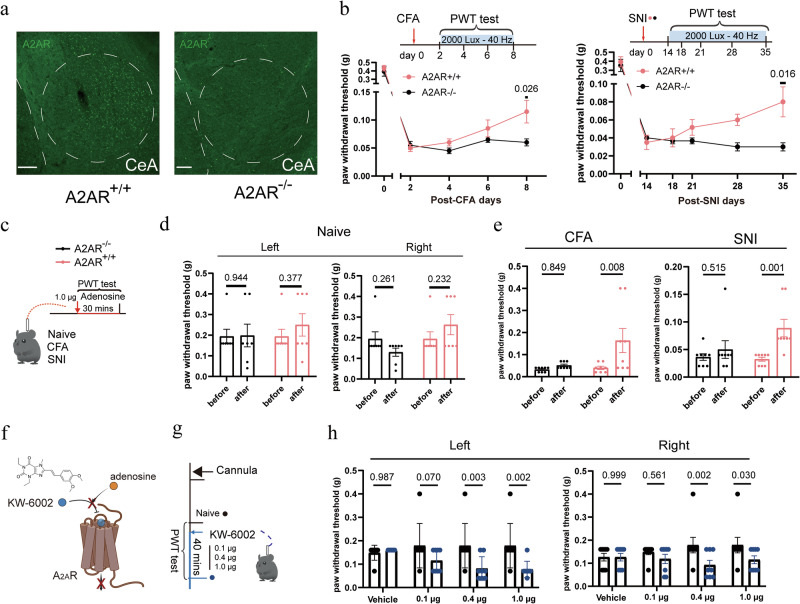
A_2A_R mediates the analgesic effects of 40 Hz light flickering and adenosine. **a** Immunostaining shows A_2A_R expression in the CeA of WT mice, which is absent in A_2A_R knockout (A_2A_R^-/-^) mice. Scale bars, 100 µm. **b** 40 Hz light flickering failed to induce antinociceptive effects in A_2A_R^-/-^ mice in both the CFA and SNI models, whereas robust analgesia was observed in their littermate controls (A_2A_R^+/+^). Error bars represent SEM. Numerical labels indicate *P* values from between-group comparisons. For behavioral tests, *n* = 6 mice in each group. **c** Schematic of intra-CeA adenosine administration in A_2A_R^-/-^ mice and their littermate controls (A_2A_R^+/+^). **d** Under naïve conditions, intra-CeA adenosine injection (1.0 μg) did not alter the PWT in either A_2A_R^+/+^ or A_2A_R^-/-^ mice. Error bars represent SEM. Numerical labels indicate *P* values from between-group comparisons. For behavioral tests, *n* = 7 mice in each group. ns, not significant. **e** In CFA and SNI models, intra-CeA adenosine (1.0 μg) significantly increased the PWT in A_2A_R^+/+^ mice but not in A_2A_R^-/-^ mice, indicating the requirement of A_2A_R for adenosine-mediated analgesia. Error bars represent SEM. Numerical labels indicate *P* values from between-group comparisons. For behavioral tests, *n* = 8 mice in each group. **f**, **g** Schematic of intra-CeA KW6002 (A_2A_R antagonist) administration. **h** Intra-CeA injections of KW6002 at doses of 0.4 μg and 1.0 μg significantly reduced the PWT in WT mice, suggesting an essential role of A_2A_R in the CeA in basal nociceptive thresholds. Error bars represent SEM. Numerical labels indicate *P* values from between-group comparisons. For behavioral tests, *n* = 8 mice in each group.

Using immunostaining, we discovered that the adenosine receptor A_2A_R was expressed in the CeA of WT mice but not in that of A_2A_R knockout (A_2A_R^‒/‒^) mice (Fig. 9a). This prompted us to investigate the contribution of A_2A_R in the context of analgesia induced by 40 Hz light flickering (Fig. 9b). Unlike littermate controls, A_2A_R-knockout (A_2A_R^‒/‒^) mice failed to respond to 40 Hz light flickering to produce analgesia in both pain models (Fig. 9b), confirming that this receptor subtype is essential for the analgesic effects. To further isolate the CeA as the locus of adenosine action, we performed intra-CeA adenosine infusion in A_2A_R^‒/‒^ mice and their WT littermate controls (Fig. 9c). Adenosine failed to exert analgesic effects in the mutant mice (Fig. 9d, e), confirming a CeA-localized and receptor-specific mechanism. Moreover, administration of KW6002,^49^ a selective A_2A_R antagonist, directly into the right CeA at a dose of 0.4 μg or 1.0 μg significantly lowered the paw withdrawal threshold in naïve mice, indicating that the A_2A_R in this brain region plays a crucial role in mechanical sensitivity (Fig. 9f–h). These results suggest that 40 Hz light flickering exerts analgesic effects through adenosine activation of A_2A_R in the CeA neurons.

### Enkephalinergic neurons in CeA are essential for the analgesia induced by 40 Hz light flickering

Given the key role of the CeA in mediating 40 Hz light flickering-induced analgesia, we next sought to identify the specific neuronal subtypes that are activated by light stimulation. The CeA is a neurochemically diverse region with multiple subpopulations that contribute to sensory and affective processing.

**Fig. 10:**
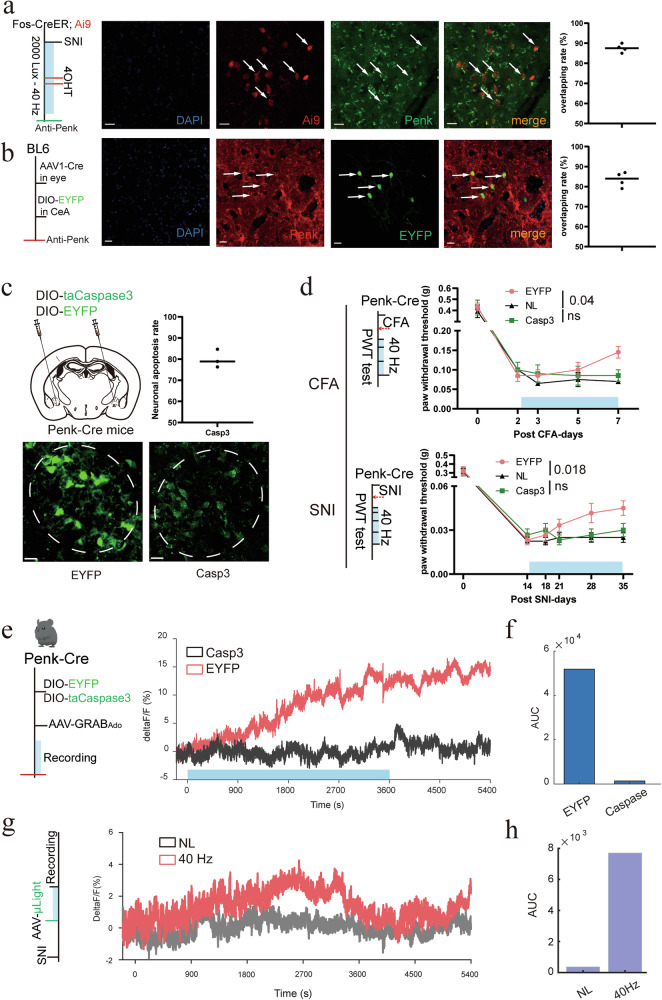
Penk^+^ neurons in the CeA mediate the analgesia and extracellular adenosine increase induced by 40 Hz light flickering. **a** Immunostaining reveals that CeA neurons activated by 40 Hz light flickering are predominantly enkephalin (Penk) positive. Scale bars, 50 μm. Error bars represent SEM. *n* = 3 mice. **b** Postsynaptic neurons within the RGC-CeA circuit are largely Penk⁺. Scale bars, 50 μm. Error bars represent SEM. *n* = 3 mice. **c** Top: schematic of Penk⁺ neuron ablation in the CeA using Cre-dependent caspase-3 expression. Error bars represent SEM. *n* = 3 mice. Bottom: representative image showing selective ablation of Penk⁺ neurons in the CeA. Scale bar, 100 μm. **d** Ablation of Penk⁺ neurons in the CeA abolished the analgesic effects of 40 Hz light flickering in both CFA and SNI mice. Error bars represent SEM. Numerical labels indicate *P* values from between-group comparisons. For behavioral tests, *n* = 6 mice in each of the EYFP and Casp3 groups, and *n* = 8 mice in the NL (no light stimulation) group. ns, not significant. **e**, **f** Penk⁺ neuron ablation in the CeA blocked the 40 Hz light flickering-induced increase in extracellular adenosine levels. *n* = 6 mice in each group. **g**, **h** 40 Hz light flickering elicited a significant increase in endogenous enkephalins in the CeA. *n* = 6 mice in each group.

To determine which CeA cell types receive direct input from RGCs, we injected the anterograde transport virus AAV1-Cre into the retina and the Cre-dependent reporter virus AAV2/9 hSyn-DIO-mCherry into the CeA. Immunohistochemistry revealed that approximately 80% of mCherry-labeled CeA neurons were immunopositive for proenkephalin (Penk), identifying postsynaptic neurons of this retina-CeA pathway as mostly enkephalinergic CeA cells (Fig. 10a, b). These cells are known for releasing enkephalins, endogenous opioid peptides that bind to opioid receptors and reduce pain.^50^ Corticotropin-releasing factor (CRF), a pro-nociceptive marker for CeA neurons, showed minimal overlap with retinorecipient CeA neurons (Supplementary information, Fig. S4a). Using Fos-CreERT2/TRAP2;Ai9 mice, we confirmed that the 40 Hz light-TRAPed CeA neurons were predominantly enkephalinergic, with > 80% co-expressing Penk (Fig. 10a, b).

To test the functional role of these neurons, we selectively ablated Penk^+^ cells in the CeA using a Cre-dependent AAV expressing the pro-apoptotic taCaspase3 in Penk-Cre mice. Histological analysis confirmed that over 80% of Penk^+^ neurons were eliminated (Fig. 10c). Importantly, ablation of these neurons abolished the analgesic effects of 40 Hz light stimulation in both CFA and SNI models (Fig. 10d). Moreover, 40 Hz light stimulation failed to induce an increase in extracellular adenosine in these mice (Fig. 10e, f), suggesting that these neurons are not only necessary for behavioral analgesia but are also required for adenosine release. Thus, Penk-expressing CeA neurons serve as a critical cellular substrate linking 40 Hz light, retinal input, adenosine signaling, and behavioral outcomes in the context of chronic pain. We then used the recently developed genetically encoded opioid peptide sensor µLight to measure endogenous enkephalin release^51^ and found that 40 Hz light flickering significantly increased enkephalin levels in the CeA (Fig. 10g, h).

We also examined adenosine A_1_R expression in the CeA. Although A_1_R is a well-known Gi/o-coupled receptor involved in pain inhibition and is considered a promising non-opioid analgesic target,^52^ our immunostaining showed that A_1_R is strongly expressed in the basolateral amygdala but only weakly expressed in the CeA (Supplementary information, Fig. S4b). By contrast, A_2A_Rs, which are Gs-coupled and generally excitatory, were more relevant in our experiments. Because 40 Hz light flickering activated downstream Penk^+^ CeA neurons to produce analgesia, these results suggest that the antinociceptive effects are more likely mediated by the excitatory A_2A_R rather than the inhibitory A_1_R.

### 40 Hz light flickering erases pain memory via the CeA

One of the key cellular processes underlying chronic pain is central sensitization.^53^ Resembling neuroplasticity in the hippocampus, central sensitization in the spinal dorsal horn enhances synaptic efficacy, leading to persistent maladaptive changes in response to painful stimuli.^15^ However, it remains unclear whether a process analogous to memory formation occurs in the CeA during pain. To address this question, we examined whether mechanical hyperalgesia could be rendered labile and reversible by targeting the CeA following re-sensitization.

**Fig. 11:**
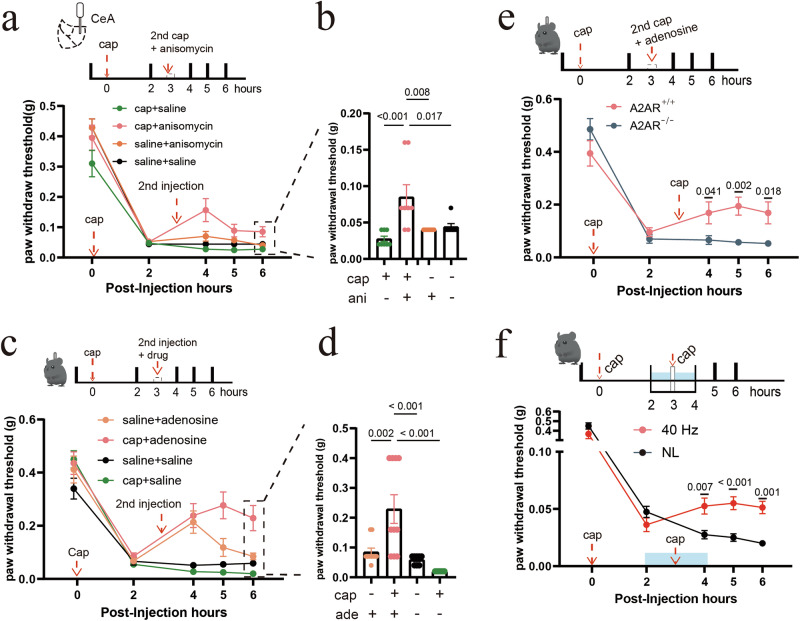
Elevated adenosine levels in the CeA destabilize pain-related memory traces. **a** Top: schematic illustrating disruption of pain memory consolidation via intra-CeA anisomycin infusion. Bottom: changes in PWT following an initial intraplantar capsaicin injection and a second identical capsaicin (or saline) injection paired with either intra-CeA saline or anisomycin. For behavioral tests, *n* = 8 mice in the capsaicin-injected group, and *n* = 7 mice in the saline-injected group. **b** Summary of the results in **a**. Error bars represent SEM. Numerical labels indicate *P* values from between-group comparisons. The results indicate that a second identical capsaicin injection paired with an intra-CeA anisomycin injection impaired the reconsolidation of capsaicin-induced hypersensitivity. **c** Top: schematic illustrating disruption of pain memory consolidation via intra-CeA adenosine infusion. Bottom: changes in PWT following a second intraplantar capsaicin injection (or saline) paired with intra-CeA infusion of either saline or adenosine. For behavioral tests, *n* = 10 mice in the capsaicin-injected group, and *n* = 8 mice in the saline-injected group. **d** Summary of the results in **c**. Error bars represent SEM. Numerical labels indicate *P* values from between-group comparisons. The results showed that a second intraplantar saline injection, paired with an intra-CeA infusion of adenosine, produced only a transient analgesia. However, a second identical capsaicin injection paired with intra-CeA infusion of adenosine elicited a significant and prolonged antinociceptive effect. **e** Top: schematic illustrating a second, identical capsaicin injection paired with intra-CeA adenosine infusion in A_2A_R^-/-^ mice and their WT littermates (A_2A_R^+/+^). Bottom: changes in the PWT when adenosine was infused into the CeA paired with a second identical capsaicin injection. For behavioral tests, *n* = 8 mice in the A_2A_R^+/+^ group, and *n* = 7 mice in the A_2A_R^-/-^ group. The results indicate that intra-CeA adenosine administration depends on A_2A_R to disrupt pain memory consolidation. Error bars represent SEM. Numerical labels indicate *P* values from between-group comparisons. **f** Top: schematic illustrating a second identical capsaicin injection paired with 40 Hz light flickering in WT mice. Bottom: changes in PWT when 40 Hz light flickering was paired with a second identical capsaicin injection. Error bars represent SEM. Numerical labels indicate *P* values from between-group comparisons. For behavioral tests, *n* = 8 mice in each group. NL, no light stimulation. The results showed that a second identical capsaicin injection paired with 40 Hz light flickering produced a robust and long-lasting analgesia, indicating disruption of pain memory.

Using an established pain memory paradigm, we induced mechanical hyperalgesia in mice via intraplantar injection of capsaicin, which produced persistent pain lasting over 6 h (Fig. 11a). Three hours after the initial capsaicin injection, the sensitized pain pathways were reactivated with a second, identical capsaicin injection, this time paired with either anisomycin (a protein synthesis inhibitor) administered into the right CeA or a vehicle control, saline. A second capsaicin injection paired with a saline injection into the right CeA had minimal effects on mechanical hypersensitivity. However, when the second capsaicin injection was co-administered with anisomycin, we observed a substantial and lasting reduction in mechanical hyperalgesia (Fig. 11a, b). These findings suggest the existence of a pain memory reconsolidation-like process in the CeA that can be disrupted by inhibiting protein synthesis.

Next, we sought to determine whether 40 Hz light flickering or adenosine signaling could interfere with pain memory in the CeA similarly to anisomycin. As shown in Fig. 11c, in the capsaicin-induced mechanical hyperalgesia model, a second injection of vehicle combined with injection of 1.0 μg adenosine into the right CeA transiently reversed mechanical hyperalgesia, but this effect diminished rapidly. By contrast, when the second capsaicin injection was directly paired with 1.0 μg adenosine in the right CeA, mechanical hyperalgesia was significantly and persistently reversed (Fig. 11c, d), an effect absent in A_2A_R^−/−^ mice (Fig. 11e). Likewise, pairing the second capsaicin injection with 40 Hz light flickering for 2 h resulted in robust and long-lasting analgesia (Fig. 11f). Together, these results demonstrate that well-established hyperalgesia can be destabilized and erased through adenosine/A_2A_R signaling in the CeA following reactivation of pain pathways.

**Fig. 12:**
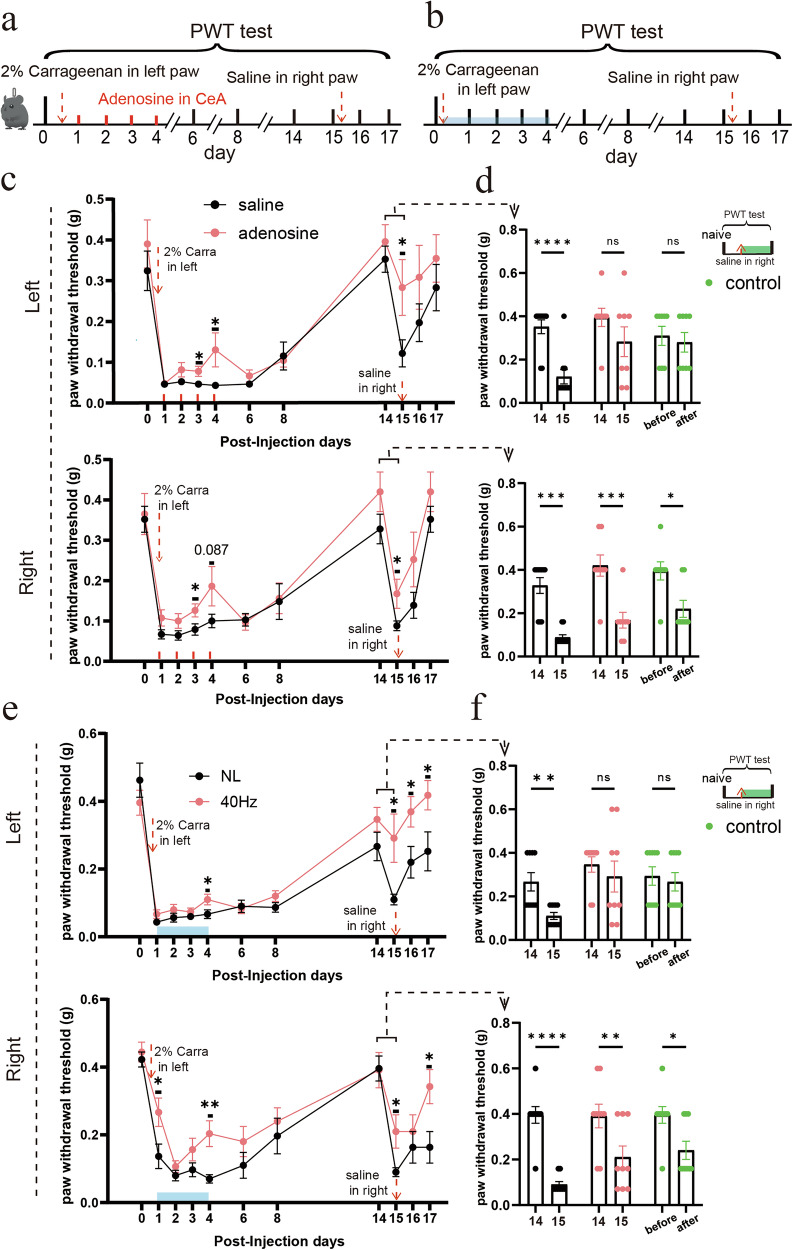
40 Hz light flickering suppresses the retrieval of pain memory. **a** Experimental design for assessing pain memory retrieval by intra-CeA adenosine infusion. **b** Experimental design for assessing pain memory retrieval by 40 Hz light flickering. **c** Changes in PWT in the carrageenan-induced inflammation model following intra-CeA adenosine infusion. Mechanical hyperalgesia was significantly reduced in both hindpaws after three days of adenosine treatment. After recovery and resolution of hypersensitivity, a crossover saline injection into the previously non-injected hindpaw triggered remote hyperalgesia in control mice, but not in the adenosine-treated group. Error bars represent SEM. Numerical labels indicate *P* values from between-group comparisons. For behavioral tests, *n* = 8 mice in the adenosine-treated group and *n* = 10 mice in the saline-treated group. ns, not significant. **d** Summary of the results in **c** after a crossover saline injection on day 15. *P < 0.05, **P < 0.01, ***P < 0.001, ****P < 0.0001; ns, not significant. Error bars represent SEM. The control group is a single saline injection performed in the right hindpaw; *n* = 8 mice. Numerical labels indicate *P* values from between-group comparisons. **e** Changes in PWT in the carrageenan-induced inflammation model following 40 Hz light flickering. Similar to adenosine treatment, 40 Hz stimulation for four consecutive days significantly alleviated mechanical hyperalgesia in both hindpaws. After recovery, a crossover saline injection evoked remote hyperalgesia in control mice, but not in the 40 Hz light-treated group. Error bars represent SEM. Numerical labels indicate *P* values from between-group comparisons. For behavioral tests, *n* = 9 mice in each group. ns, not significant. **f** Summary of the results in **e** after a crossover saline injection on day 15. *P < 0.05, **P < 0.01, ***P < 0.001, ****P < 0.0001; ns, not significant. Error bars represent SEM. The control group is a single saline injection performed in the right hindpaw; *n* = 8 mice. Numerical labels indicate *P* values from between-group comparisons.

To further investigate the influence of adenosine and 40 Hz light flickering on pain memory retrieval, we assessed distant hyperalgesia induced by a crossover vehicle injection after carrageenan-induced inflammation (Fig. 12a, b). In the mouse model, unilateral carrageenan injection triggered hyperalgesia in the injected left hindpaw that persisted for approximately one week before resolving by day 14. Concurrently, secondary hyperalgesia developed in the contralateral (right), non-injected hindpaw. In the treatment group, 1.0 μg adenosine was administered into the right CeA daily for four consecutive days following carrageenan injection, whereas the control group received vehicle-saline infusions. After four adenosine administrations, we observed a significant reduction in mechanical hyperalgesia in both the injected and non-injected hindpaws (Fig. 12c). Once the paw withdrawal threshold returned to baseline by day 14, a crossover saline injection was administered into the previously non-injected (right) hindpaw on day 15 (Fig. 12c). This elicited renewed mechanical hyperalgesia in the saline-injected hindpaws of both groups on day 15 (Fig. 12c, d). Importantly, in the previously carrageenan-injected left hindpaw, mechanical hyperalgesia re-emerged only in the saline-treated group, not in the adenosine-treated group (Fig. 12c, d). As a control, naïve mice received a single saline injection in the right hindpaw, which produced mechanical hyperalgesia only on the ipsilateral side within 4 h. No significant changes in paw withdrawal threshold were observed on the contralateral side, indicating that saline injection alone does not induce mechanical hyperalgesia in the non-injected hindpaw (Fig. 12d). These results suggest that adenosine infusion into the right CeA following initial carrageenan inflammation effectively suppresses the retrieval of pain memory.

We next examined the effect of 40 Hz light flickering using the same carrageenan-induced pain model. Following carrageenan injection, the treatment group was exposed to 2 h of 40 Hz light flickering daily for four consecutive days, whereas the control group remained under normal lighting conditions. By day 4, light treatment significantly alleviated mechanical hyperalgesia in both the injected and non-injected hindpaws (Fig. 12e). Once the paw withdrawal threshold had normalized by day 14, a crossover saline injection was administered into the previously non-injected (right) hindpaw on day 15. This procedure induced mechanical hyperalgesia in the saline-injected hindpaws of both groups on day 15. Notably, in the previously carrageenan-inflamed left hindpaw, mechanical hyperalgesia reappeared only in the untreated group; no such hypersensitivity was observed in the 40 Hz light-treated group (Fig. 12e, f). Intriguingly, in the treatment group, the paw withdrawal threshold of the previously carrageenan-injected hindpaw exhibited a transient decrease following the crossover saline injection but subsequently rebounded, continuing to rise over time (Fig. 12e). These findings indicate that 40 Hz light flickering not only alleviates ongoing pain but may also actively disrupt or reverse maladaptive pain memory traces.

## Discussion

Chronic pain is a prevalent, complex, and debilitating condition that remains difficult to treat.^2^ The maintenance of chronic pain involves both peripheral sensitization and long-term plasticity within spinal, subcortical, and cortical circuits,^15,17^ leading to heightened pain perception (hyperalgesia), pain in response to non-painful stimuli (allodynia), and the spreading of pain beyond the site of injury.^8^ These processes also contribute to the emotional and cognitive dimensions of pain, which are particularly resistant to conventional analgesics.^17^ Thus, targeting central mechanisms, especially brain regions that integrate nociceptive, affective, and cognitive aspects of pain, offers a more comprehensive and durable strategy for pain relief. Whereas current pharmacological approaches often have limited efficacy or serious adverse effects, neuromodulation, such as repetitive brain stimulation, has shown the potential to reshape maladaptive central circuits without systemic side effects.^54,55^

In this context, one of the key findings of our study is the identification of 40 Hz light flickering as a non-invasive and robust intervention for alleviating chronic pain, adding a new dimension to 40 Hz light-based neuromodulation. We demonstrated that: (i) 40 Hz flickering light significantly reduced pain behaviors in rodent models of both chronic inflammatory and neuropathic pain; (ii) this analgesic effect was dependent on both the frequency and intensity of the flickering light; (iii) 40 Hz light flickering exerted its analgesic effects by activating a direct retina-CeA pathway, as shown through circuit-specific manipulations; and (iv) 40 Hz flickering light promoted localized adenosine release within the CeA, revealing a novel, circuit-targeted mechanism that bypasses systemic adenosine exposure, thereby avoiding cardiovascular and other common side effects. Collectively, our findings establish a new, non-invasive therapeutic strategy to directly target the CeA, a key hub in pain processing, for the treatment of chronic pain. We not only delineated the neural circuitry underlying the analgesia induced by 40 Hz flickering light but also revealed its neurochemical basis. A single 1-h session of 40 Hz light flickering robustly elevated extracellular adenosine levels in the CeA, indicating a rapid and acute biochemical effect. By contrast, in both the CFA inflammatory pain model and the SNI neuropathic pain model, prolonged and repeated light stimulation was required to achieve significant analgesia. This apparent discrepancy likely reflects fundamental differences between acute neuromodulatory responses and the persistent maladaptive plasticity that sustains chronic pain. Accordingly, sustained 40 Hz light flickering may be necessary to drive progressive remodeling and reconstruction of entrenched pathological neuroplasticity in these higher brain centers, such as the CeA, ultimately producing durable analgesic effects.

Our findings indicate that the retina-CeA pathway serves a beneficial role under chronic pain conditions. Although an ipRGC-CeA circuit has previously been associated with anxiogenic responses to acute bright light,^56^ these results show that the functional effect and plasticity of retinal-brain circuits are highly dependent on both the nature of the light stimulation and the underlying pathological state. Through anatomical tracing and functional mapping, we found that RGCs project directly to enkephalin-expressing neurons in the CeA, a key node in processing emotions and aversive learning.^20^ Chronic activation of the retinorecipient CeA neurons mimicked the antinociceptive effects of 40 Hz flickering light, whereas silencing these CeA-projecting RGCs or ablation of CeA enkephalinergic neurons abolished the analgesic effects, confirming the necessity and sufficiency of this circuit. Furthermore, we demonstrated that adenosine signaling through A_2A_R in the CeA is a critical mediator of 40 Hz-induced analgesia. We observed that 40 Hz light flickering dramatically increased extracellular adenosine levels in the CeA. Local pharmacological activation of A_2A_R by adenosine recapitulated this analgesia, whereas A_2A_R-knockout mice failed to respond to either adenosine infusion or 40 Hz light stimulation. These results suggest that 40 Hz flickering light engages the retina-CeA pathway to promote endogenous adenosine release, which in turn activates A_2A_R to suppress pain.

Adenosine is a well-established modulator of both pain and synaptic plasticity. Intravenous or intrathecal administration of adenosine has been shown to alleviate allodynia and hyperalgesia in both chronic pain patients and rodent models.^32,34^ Acupuncture, a millennia-old analgesic practice, induces localized adenosine release in mice, with its antinociceptive effects entirely dependent on A_1_R activation,^57^ reinforcing the link between adenosine and endogenous pain relief. The role of A_2A_R in pain, however, is more nuanced, with evidence supporting both pronociceptive and antinociceptive actions depending on the context.^34,58^ Notably, intrathecal administration of A_2A_R agonists reversed allodynia and hyperalgesia in neuropathic pain models,^59^ consistent with our findings. The therapeutic potential of targeting adenosine receptors is widely recognized. However, a major obstacle to the clinical translation of adenosine-based therapies is the ubiquity of adenosine signaling. Activation of these receptors elicits a wide range of physiological and pathophysiological effects, contributing to the side effects that have hampered the success of adenosine receptor agonists and antagonists in clinical trials. One possible solution is to enhance the levels of endogenous adenosine locally, rather than administering exogenous ligands systemically. In this study, 40 Hz light flickering was revealed as a non-invasive strategy that enables temporally precise and spatially targeted modulation of endogenous adenosine release at the circuit level. By leveraging the specific retina-CeA pathway, this method offers a novel route for engaging adenosine signaling in a localized and controlled manner, thereby addressing one of the major limitations of systemic adenosine-based therapies.

The CeA is a highly heterogeneous structure that integrates emotion, motivation, and pain.^60,61^ Although largely GABAergic, CeA is composed of multiple genetically and functionally distinct populations of neurons, most notably including subtypes that express protein kinase C delta (PKCδ**)**, somatostatin (SST), and CRF. These subpopulations exert opposing control over pain-related behaviors. For example, one study showed that PKCδ^+^ neurons are sensitized by nerve injury and promote nocifensive responses, whereas SST^+^ neurons are inhibited by nerve injury and drive antinociception,^62^ although others have reported conflicting results.^63^ It is plausible that these pain-associated PKCδ^+^ neurons could be inhibited by enkephalin released from retinorecipient CeA neurons activated by adenosine-A_2A_R signaling, although this possibility will require future experimental validation. Interestingly, general anesthetics engage a common neuronal ensemble in the CeA that potently suppresses pain,^64,65^ with a large fraction of activated neurons expressing Penk, suggesting potential overlap with the retinorecipient Penk^+^ population identified in our study. The CeA also contains a high density of CRF-expressing neurons.^60^ CRF expression in the CeA is elevated in pain models, and knockdown of CRF in the CeA reversed persistent visceral and somatic hyperalgesia.^66^ Consistent with this pro-nociceptive role, a substantial proportion of CRF neurons receive input from CGRP-expressing afferents that arise from the parabrachial nucleus.^67^ Importantly, our results show that retinorecipient CeA neurons largely do not express CRF, indicating the cell-type specificity of the retina-CeA pathway. In addition, CeA receives direct nociceptive input from the spino-parabrachio-amygdaloid pathway.^68,69^ Recent studies further demonstrated that CeA neurons are involved in encoding threat memories via CGRP-expressing parabrachial inputs.^70^ Notably, inhibition of CGRP neurons abolished escape behaviors without affecting reflexive pain responses, highlighting the separable sensory and affective components of pain in the CeA.^70^ In testing the existence of pain memory in the CeA, we found that capsaicin-induced mechanical hyperalgesia became labile upon reactivation and could be reversed by intra-CeA injection of the protein synthesis inhibitor anisomycin — a phenomenon reminiscent of hippocampus-dependent memory reconsolidation. Strikingly, adenosine infusion into the CeA produced long-lasting analgesia when paired with reactivation of CeA pain pathways via a second, identical capsaicin injection, mimicking the effects of anisomycin. Crucially, these effects were dependent on A_2A_R signaling, as A_2A_R-knockout mice showed no such therapeutic response. These memory-disrupting effects were recapitulated by 40 Hz light flickering, suggesting that chronic gamma-frequency stimulation can functionally rewrite maladaptive pain memories. In a separate carrageenan-induced inflammation model, we revealed memory-like properties: mechanical hyperalgesia in a previously inflamed hindpaw could be recalled by a remote contralateral saline injection even after resolution of the original inflammation. Both intra-CeA adenosine infusion and 40 Hz light flickering suppressed this memory retrieval, significantly reducing the recalled hyperalgesia. These findings suggest that neuromodulatory pathways in the CeA can be harnessed to rewrite maladaptive pain-related plasticity. Given our finding that adenosine signaling in the CeA suppresses pain memory, this CGRP-expressing parabrachial-CeA input is well positioned to be gated by changes in extracellular adenosine during 40 Hz stimulation.

Although our data demonstrate a critical role of the CeA in mediating the modulation of pain memory by 40 Hz light flickering, pain memory is widely recognized as a distributed process that involves multiple interconnected cortical and subcortical regions. The anterior cingulate cortex is essential for the affective-motivational dimension of pain and for the persistence of aversive pain memory through long-term synaptic plasticity mechanisms.^71^ The prefrontal cortex contributes to pain expectation, cognitive evaluation, and top-down regulation of pain-related memory and behavior.^72^ Importantly, hippocampal neurons undergo robust structural and functional plasticity in chronic pain states, reflecting the formation of persistent nociceptive memory traces.^73^ Thus, the CeA-based modulatory system identified in this study could be embedded within a broader, distributed pain memory network.

As a ubiquitous signaling molecule, adenosine acts as a bidirectional modulator of synaptic plasticity, with its effects depending on receptor subtypes, brain regions, and activity state. Its ability to fine-tune synaptic strength makes it crucial for learning, memory, and neuroprotection.^58^ A_1_R generally inhibits LTP and impairs memory,^74–76^ whereas A_2A_R has context-dependent roles. A_2A_R facilitates LTP at hippocampal mossy fiber synapses,^77,78^ but excessive A_2A_R activation impairs memory and is implicated in aging and Alzheimer’s pathology.^79–81^ In our study, CeA-targeted A_2A_R activation through adenosine or 40 Hz light flickering selectively disrupted pain memory, highlighting its therapeutic specificity.

In conclusion, by demonstrating that chronic pain is actively maintained through memory-like mechanisms within the CeA, our findings fundamentally reframe the understanding of pain persistence. We leveraged a direct retina-brain pathway through which 40 Hz light flickering reprogrammed maladaptive pain circuits via adenosine-A_2A_R signaling, revealing that this pathway can mitigate hyperalgesia and disrupt established pain memory. This convergence of visual stimulation and endogenous adenosine signaling in the CeA not only provides a mechanistic explanation for the analgesic effects of 40 Hz stimulation but also opens new avenues for non-invasive, circuit-specific, and neurochemically targeted therapies. These results lay the groundwork for a paradigm shift in the treatment of chronic pain — one that embraces the interplay among sensory experience, memory, and neuromodulation.

## Materials and methods

### Animals

C57BL/6 mice were purchased from Charles River Laboratories. TRAP2/Fos 2A-iCreER (Stock #030323), Ai9 (Stock #007909), and Penk-Cre (Stock #025112) mice were obtained from The Jackson Laboratory. A_2A_R^−/−^ mice were generated and bred in-house as described previously.^82^ The Fos 2A-iCreER mouse was crossed with the Ai9 mouse to generate the Fos-CreER;Ai9 mouse line. Mice were housed under a standard light/dark cycle (12 h:12 h, 23 °C–25 °C) with free access to food and water. Animal experiments were conducted according to protocols approved by the Institutional Animal Care and Use Committee of Wenzhou Medical University.

### Pain models

For the CFA model, male mice were briefly anesthetized with isoflurane, and a subcutaneous injection of 20 μL of 50% CFA (Sigma) was administered into the plantar surface of the hind paw. SNI was used to establish the neuropathic pain model. Male mice were anesthetized with 1.2% avertin (2,2,2-tribromoethanol/tert-amyl alcohol, Sigma). The skin on the lateral surface of the thigh was incised, and a section was made directly through the biceps femoris muscle to expose the sciatic nerve and its three terminal branches: the sural, common peroneal, and tibial nerves. The SNI procedure comprised axotomy and ligation of the tibial and common peroneal nerves, leaving the sural nerve intact. The common peroneal and tibial nerves were tightly ligated with 5.0 silk and sectioned distal to the ligation, removing about 2 mm of the distal nerve stump. The incision was sutured and disinfected. Great care was taken to avoid any contact with or stretching of the intact sural nerve. For the spontaneous pain model, 10 μL of 5% formalin (catalog no. 252549, Sigma) was injected subcutaneously into the left whisker pad. Scratching behaviors were recorded, with Phase I defined as 0–5 min and Phase II as 20–40 min after the formalin injection.

### Pain memory models

Two distinct pain memory models were used in this study.

#### Capsaicin-induced mechanical hyperalgesia

Male mice received a 5-μL subcutaneous injection of 0.5% capsaicin (prepared in 10% ethanol/90% saline) into the left hindpaw. Three hours later, a second injection of either identical capsaicin or vehicle was administered to the same hindpaw to reactivate the memory trace. Simultaneously, 40 Hz light flickering or pharmacological agents (2 μL total volume) were infused into the CeA via implanted cannulae at a rate of 0.5 μL/min. The agents included adenosine (0.5 μg/μL, pH adjusted to 7.3), anisomycin (20 μg/μL, pH adjusted to 7.3), and corresponding vehicle controls. Mechanical sensitivity was assessed at multiple time points (2 h, 4 h, 5 h, and 6 h post-reactivation) to distinguish transient pharmacological effects from disruption of memory reconsolidation.

#### Carrageenan-induced inflammation

The second model utilized carrageenan-induced inflammation in male mice. The initial inflammatory response was triggered by a subcutaneous injection of 10 μL of 2% carrageenan (Sigma, USA) into the plantar surface of the right hindpaw. Fourteen days later, the plantar surface of the left hindpaw received a saline injection. Nociceptive memory was evaluated by measuring distant hyperalgesia, wherein the previously injected right hindpaw was not reinjected when a crossover saline injection was administered to the left hindpaw.

### Behavioral test

Male mice were habituated to the testing chambers for at least 2 h before behavioral assessments. According to the up-down method,^83^ a series of calibrated von Frey filaments (0.02–2.0 g, Anesthesio) were applied to the plantar surface of the hind paws to determine the withdrawal threshold. Each filament was used five times at an interval of a few seconds. When paw withdrawal reactions were elicited in more than 50% of trials, the mouse was considered to have mechanical sensitivity to the filament force used. All behavioral tests were performed blindly. To measure paw withdrawal frequency, von Frey filaments (0.02–0.4 g, Anesthesio) were each applied 10 times to the plantar surface of the hind paws, and the number of withdrawal responses was recorded. Noxious thermal sensation was assessed using a hot and cold plate (Bioseb). Cold sensitivity was measured by recording the paw withdrawal latency on a 4 °C plate. For heat sensitivity, mice were tested on a 55 °C hot plate, and hind paw withdrawal latency was recorded. A cutoff time of 20 s was set to prevent tissue injury.

### Light flickering stimulation

Light flickering stimulation was performed as described previously.^38,84^ Before light stimulation, male mice were acclimated for 30 min in transparent chambers mimicking home cage conditions (with minimal bedding) under dim ambient light. Two white LED panels (CCT at 4000 K, 390–700 nm spectrum) were positioned on opposite chamber walls and controlled by a relay system to deliver various stimulation paradigms: light and 20/40/80 Hz pulsed light at 1000/2000/4000 lux intensities (≤ 1.5 mW/cm² irradiance, verified by dosimetry). Two exposure regimens were implemented from 08:00 to 20:00 daily: (1) short exposure: 1 h illumination every 12 h (total 2 h/day), and (2) long exposure: two 4-h illumination sessions in the morning and evening, respectively (total 8 h/day). Both protocols maintained identical environmental controls (22°C ± 0.5 °C) and included continuous safety monitoring.

### Stereotaxic injection

Male mice were anesthetized with 1.2% avertin and head-fixed with a stereotaxic frame (RWD, China). Ophthalmic sterile ointment was applied to prevent corneal drying. Viruses were injected using a glass micropipette attached to the Nanoject III Programmable Nanoliter Injector (#3-000-207, DRUMMOND). AAVs were injected at a flow rate of 2 nL/s at the following stereotaxic coordinates: CeA (AP: –1.35 mm from bregma; ML: ± 2.87 mm; DV: –4.50 mm). Following the injection, the needle was left in place for 5 min post-injection to prevent backflow. The following AAV viruses were used: scAAV1-hSyn-Cre (S0292-1, Taitool Bioscience, China), AAV2/2Retro Plus-hSyn-Cre (S0278-2RP, Taitool Bioscience, China), AAV2/2Retro Plus-hSyn-Cre-EGFP (S0230-2RP, Taitool Bioscience, China), AAV2/9-hSyn-DIO-hM3D(Gq)-mCherry (PT-0019, BrainVTA, China), AAV2/9-hSyn-DIO-hM4D(Gi)-mCherry (PT-0020, BrainVTA, China), AAV2/9-hSyn-DIO-mCherry (PT-0115, BrainVTA, China), AAV2/9-EF1α-DIO-hChR2(H134R)-EYFP (PT-0001, BrainVTA, China), AAV2/9-EF1α-DIO-EYFP (PT-0012, BrainVTA, China), AAV2/9-EF1α-DIO-TeNT-EGFP (PT-2434, BrainVTA, China), AAV2/9-EF1α-DIO-taCasp3-TEVp-EGFP (PT-1230, BrainVTA, China), AAV2/9-hSyn-Ado_B10 (PT-1348, BrainVTA, China), AAV2/9-hSyn-Ado_B10mut (PT-1349, BrainVTA, China), AAV2/9-hSyn-DIO-hM3D(Gq)-EGFP (PT-0891, BrainVTA, China), AAV2/8-EF1α-DIO-H2B-EGFP-T2A-TVA (PT-0021, BrainVTA, China), AAV2/8-EF1α-DIO-oRVG (PT-0023, BrainVTA, China), RV-EnvA-ΔG-mCherry (R01004, BrainVTA, China), and AAV2/9-hSyn-δlight (PT-11760, BrainVTA, China). For RV-based transsynaptic tracing, 60 nL of AAV2/8-EF1α-DIO-oRVG and 60 nL of AAV2/8-EF1α-DIO-H2B-EGFP-T2A-TVA were mixed and co-injected into the CeA. Twenty-one days later, 120 nL of RV-EnvA-ΔG-mCherry was injected into the same site.

### Intravitreal injection

Ocular injections required specialized techniques to achieve intraocular delivery while preserving retinal integrity. Male mice were anesthetized as described previously, with additional topical application of 0.5% proparacaine to the cornea for local analgesia. Under a surgical microscope, the globe was gently protruded using curved forceps positioned at the lateral canthus, exposing the pars plana region. A 33-gauge needle attached to a dual-channel microinjection system was inserted 1 mm posterior to the limbus at a 30° angle, ensuring that the lens was not contacted. Following a 60-s equilibration period to allow PBS egress via the trabecular meshwork, 900 nL of viral suspension was injected through the same needle track. The injection volume did not exceed 4% of the total vitreous volume (∼50 µL in adult mice) in order to prevent retinal detachment. Transient corneal clouding was an expected immediate post-procedural observation and typically resolved within 24 h.

### Cannula implantation and administration of drugs

To administer drugs into the CeA, 26-gauge stainless steel guide cannulas (KedouBC, China) were implanted into the CeA using a microdriver and then affixed with dental cement anchored by two skull screws. A tubing-nested 10-μL Hamilton syringe was used for drug microinjections. All pharmacological agents were delivered via an indwelling cannula implanted in the right CeA (stereotaxic coordinates: AP – 1.35 mm, ML – 2.87 mm, DV – 4.45 mm from bregma). Adenosine (A9251, Sigma) was dissolved in sterile saline and applied at three different doses: 0.2 μg, 1.0 μg, and 2.0 μg. Dipyridamole (D9766, Sigma) was first prepared as a 200 mg/mL stock solution in DMSO (protected from light at –20 °C) and then diluted in corn oil to final concentrations of 0.1 mg/mL, 0.5 mg/mL, and 2 mg/mL immediately before use. For KW6002 (HY-10888, MCE) administration, a 1:1 DMSO/castor oil mixture was used to dissolve the compound into a 5 mg/mL stock solution, which was further diluted with saline to working concentrations (0.05 mg/mL, 0.2 mg/mL, and 0.5 mg/mL) and vortexed for 30 s prior to injection. Anisomycin (HY-18982, MCE) was dissolved in 3 N HCl at 37 °C with continuous gentle agitation for 10 min to ensure complete solubilization. The acidic solution was then neutralized to pH 7.3 ( ± 0.1) by dropwise addition of 3 N NaOH, monitored using a calibrated micro-pH meter (Thermo Scientific). The neutralized solution was adjusted to a final concentration of 20 μg/μL by dilution with sterile saline. All drug solutions were infused at a volume of 2 μL over 2 min using a micro infusion pump (KD Scientific, USA), with the tubing-nested 10-μL Hamilton syringe left in place for an additional 5 min to prevent backflow.

### Fiber photometry

The fiber photometry system for monitoring extracellular adenosine dynamics in the CeA began with stereotaxic delivery of AAV2/9-hSyn-GRABAdo or AAV2/9-hSyn-δlight combined with implantation of an optical fiber (200 µm core diameter, 0.37 NA). Under isoflurane anesthesia (1.5%–2% maintenance), the mouse head was fixed in a stereotaxic frame with bregma-lambda alignment confirmed within ±0.05 mm. After the skull was exposed and bilateral burr holes targeting CeA coordinates (AP: –1.35 mm, ML: ± 2.87 mm, DV: –4.50 mm from bregma) were drilled, 250 nL viral solution was infused at 50 nL/min via a glass micropipette attached to a Nanoject III Programmable Nanoliter Injector (#3-000-207, DRUMMOND), followed by a 10-min diffusion wait step. A ceramic ferrule-terminated black fiber optic was then lowered to –4.40 mm DV and secured with dental cement, and the wound was closed with sutures. After three weeks of incubation for GRABAdo or enkephalin sensor expression, mice were habituated to the photometry chamber for 3 consecutive days (30 min/day) to minimize movement artifacts.

Adenosine or enkephalin signals were recorded using a commercial fiber photometry system (Thinker Tech Nanjing Biotech Co., Ltd., China). To ensure artifact-free fiber photometry recordings during concurrent external light stimulation, cosmetic-grade nail polish was applied to the dental cement surrounding the implanted fiber optic, preventing ambient light leakage into the fiber-optic pathway and blocking cross-talk from external optogenetic stimulation sources. To record fluorescence signals on the experiment day, the beam from a 470-nm laser (OBIS 470LS; Coherent) was reflected by a dichroic mirror (MD498; Thorlabs), focused by a 10× objective lens (NA = 0.3; Olympus), and then coupled to an optical commutator (Doric Lenses). Fiber recordings were performed on freely moving mice during flicker visual stimulation, and no data were excluded. The mice were connected to the fiber photometry recording system, and the signals were recorded for 1200 s as a baseline before flicker visual stimulation. The fluorescence signals from each experimental trial were normalized using a MATLAB-based analysis pipeline developed by Thinker Tech. Raw data underwent photobleaching correction by detrending the signal baseline prior to further processing. Adenosine/ATP or enkephalin response magnitudes to flicker stimulation were quantified as ΔF/F values, calculated as (F − F_0_)/(F_0_ − V_offset_), where F_0_ represents the 200-s pre-stimulus baseline average. Peri-event time histograms were generated with polynomial baseline correction to visualize ΔF/F dynamics. Experimental validity was confirmed through parallel recordings using a multicolor single-channel system. Quantitative analysis involved measuring the area under ΔF/F curves during stimulation epochs, with the results presented as mean peri-event plots (± SEM). To avoid any potential effects of anesthetics on adenosine or enkephalin release, all fiber photometry recordings were performed in freely moving animals without the use of anesthesia.^85^

### Histology and immunohistochemistry

#### Tissue preparation and cryosectioning

Mice were deeply anesthetized with isoflurane (5% for induction, 1.5%–2% for maintenance) and transcardially perfused with ice-cold 0.1 M PBS, followed by 4% paraformaldehyde (PFA) for fixation. Brains were then removed and post-fixed in 4% PFA at 4 °C for 18 h. After fixation, tissues were cryoprotected in 30% sucrose in PBS at 4 °C until they sank (24–48 h), embedded in OCT compound, frozen on dry ice, and stored at –80 °C. Coronal brain sections (25 μm thick) were cut using a cryostat (Microm HM 550, Thermo Fisher Scientific) at –20 °C and stored free-floating in cryoprotectant at –80 °C.

#### Immunohistochemistry

Sections were incubated at room temperature in PBS for 15 min and then washed twice with PBS (5 min each). They were permeabilized in 0.5% Triton X-100/PBS (PBST) for 30 min and blocked in 5% BSA/PBS for 1 h at room temperature with gentle shaking. Primary antibodies diluted in 5% BSA/PBS were applied overnight at 4 °C with continuous agitation. The next day, sections were washed three times with PBST (10 min each) and incubated with species-specific secondary antibodies diluted in 1% BSA/PBS for 2 h at room temperature in the dark. After three final PBS washes (10 min each), DAPI (0.02 mg/mL) was applied for 1 min. Sections were mounted on Superfrost Plus slides using fluorescence-compatible mounting medium and stored at 4 °C in the dark until imaging. For Penk labeling, rabbit anti-Penk (1:200, K004421P, Solarbio) was applied for 48 h at 4 °C, followed by goat anti-rabbit Alexa Fluor 488 (1:1000, A11008, Invitrogen) for 2 h at room temperature. For A_2A_R labeling, goat anti-A_2A_R (1:500, A2A-Go-Af700, Frontier Institute) was applied for 24 h at 4 °C, followed by donkey anti-goat Alexa Fluor 488 (1:1000, A11055, Invitrogen) for 2 h at room temperature. For A_1_R labeling, rabbit anti-A_1_R (1:500, 20332-1-AP, Proteintech) was applied for 24 h at 4 °C, followed by goat anti-rabbit Alexa Fluor 568 (1:1000, A11011, Invitrogen) for 2 h at room temperature. For CRF labeling, rabbit anti-CRF (1:200, 10944-1-AP, Proteintech) was applied for 24 h at 4 °C, followed by goat anti-rabbit Alexa Fluor 568 (1:1000, A11011, Invitrogen) for 2 h at room temperature. Fluorescence images were acquired using a Leica confocal microscope. Image analysis, including cell counting and colocalization, was performed using ImageJ.

#### Retina whole-mount immunostaining

Mouse eyes were enucleated following euthanasia and fixed in 4% PFA for 10 min at room temperature, then post-fixed at 4 °C for 1 h. Retinas were dissected, flattened into four quadrants, and permeabilized in 0.5% Triton X-100 for 2 h at room temperature. After PBS washes, samples were blocked and incubated with rabbit anti-melanopsin (1:1000, AB-N39, Advanced Targeting Systems) overnight at 4 °C. Following additional washes, retinas were incubated with goat anti-rabbit Alexa Fluor 568 (1:1000, A11011, Invitrogen) for 2 h at room temperature. After final washes, tissues were mounted flat on Superfrost Plus slides for imaging.

### In vivo electrophysiology

Male mice (8–10 weeks old) underwent aseptic surgery for chronic local field potential (LFP) electrode implantation targeting the CeA. Under isoflurane anesthesia (induction: 3%–4%, maintenance: 1.5%–2% in O₂), animals were secured in a stereotaxic frame (RWD, China) with bregma-lambda alignment verified within ±0.05 mm. After scalp disinfection (povidone-iodine/70% ethanol), a midline incision exposed the skull, and a unilateral cranial window was drilled (CeA coordinates: AP – 1.35 mm, ML ± 2.87 mm relative to bregma). A 16-channel (4 × 4 array) microwire electrode (Kedou KD-MWA-16) was slowly lowered to DV – 4.50 mm from the dura at 1 mm/s. Two bone screws placed above the cerebellar hemispheres served as ground/reference. The assembly was secured by sequential application of tissue adhesive and dental cement, ensuring < 0.5 MΩ impedance across channels.

Following a 7-d postoperative recovery period, mice were placed under carefully titrated light anesthesia (1%–1.2% isoflurane in 30% O₂/70% N₂) while maintaining stable respiratory rates (90–110 bpm) and preserved corneal reflexes, then secured in a stereotaxic frame with incisor bar positioning optimized to minimize motion artifacts. The recording system was connected via a 16-channel digitally programmable amplifier (0.1 Hz–10 kHz), and a high-intensity LED array (2000 lux at 15-cm distance) was precisely positioned anterior to the mouse snout within an electromagnetically shielded copper mesh cage (λ/4 at 50 Hz) to eliminate environmental interference. Baseline LFP recordings were acquired for 2 min under dark adaptation (0.1 lux) before initiating the 3-min visual stimulation protocol (40 Hz); negative controls included electrodes buried in saline and reverse-positioned LEDs (illumination directed away from the mouse).

The LFP data analysis pipeline was implemented in MATLAB R2017b with rigorous spectral processing standards. Raw signals were first down-sampled to 1 kHz, followed by short-time Fourier transform with 500-ms Hanning windows (75% overlap) to generate time-frequency spectrograms. Notch filters (4th-order Butterworth) were systematically applied to eliminate 50 Hz line noise and its harmonics (100 Hz, 150 Hz, etc.), achieving > 90% interference suppression as verified by power spectral density comparisons. The 40 Hz gamma-band power was quantified by averaging spectral power within a narrow 39.7–40.3 Hz bandwidth.

### HPLC analysis of total adenosine in the CeA

HPLC assays were performed as described previously.^38^ Mice were randomly assigned to three groups: naïve mice under normal light, SNI mice, and SNI mice exposed to 40 Hz light flickering. Following a 1-h light flickering session, mice were euthanized by rapid decapitation. The CeA was rapidly dissected and stored at –80 °C until analysis. To precipitate proteins, CeA samples were mixed with a precooled solution containing adenosine inhibitors (500 µL of 1 mg/mL dipyridamole and 20 µL of 20 µg/mL EHNA) and acetonitrile at a 1:19 (v/v) ratio. Samples were vortexed for 30 s and centrifuged at 12,000 rpm for 15 min. The supernatant (100 µL) was collected and injected into the HPLC system for analysis. Adenosine was separated using a Polaris 5 C18-A column (Agilent; 2.1 mm × 150 mm, 5 µm) maintained at 25 °C, with detection at 258 nm. Isocratic elution was performed at a flow rate of 1 mL/min using a mobile phase consisting of 0.1% methanol (phase A) and 99.8% phase B. Phase B was prepared with 25 mM sodium dihydrogen phosphate and adjusted to pH 7.0 with triethylamine.

### Electrophysiology of brain slices

#### CeA slice preparation

Acute brain slices from adult C57BL/6 mice expressing ChR2 or EYFP were obtained as described previously.^86^ In brief, mice were deeply anesthetized with 1.25% avertin and transcardially perfused with ice-cold oxygenated (95% O_2_/5% CO_2_) *n*-methyl-*d*-glucamine (NMDG)-based cutting solution (93 mM NMDG, 2.5 mM KCl, 1.2 mM NaH_2_PO_4_, 30 mM NaHCO_3_, 25 mM glucose, 20 mM HEPES, 5 mM sodium ascorbate, 3 mM sodium pyruvate, 10 mM MgSO_4_, 0.5 mM CaCl_2_; pH 7.3–7.4 and 300–310 mOsmol). Coronal sections (300 μm) containing the CeA were prepared using a VT1200 S vibratome (Leica) under continuous oxygenation. Slices were initially recovered in NMDG-artificial cerebrospinal fluid (ACSF) at 34 °C for 15 min, then transferred to another oxygenated ACSF (92 mM NaCl, 2.5 mM KCl, 1.2 mM NaH_2_PO_4_, 26 mM NaHCO_3_, 20 mM HEPES, 25 mM glucose, 2 mM CaCl_2_, 2 mM MgSO_4_, 3 mM sodium pyruvate, and 5 mM sodium ascorbate; 300–310 mOsmol and pH 7.3–7.4) for 1 h and maintained at room temperature until recording. All chemicals for the preparation of slices were obtained from Sigma.

#### Electrophysiological recordings of retinorecipient CeA neurons

Following recovery, the slices were placed in the recording chamber and continuously perfused with ACSF (124 mM NaCl, 1.2 mM NaH_2_PO_4_, 2.5 mM KCl, 2 mM MgSO_4_, 12.5 mM glucose, 2 mM CaCl_2_, and 24 mM NaHCO_3_; pH 7.3–7.4 and 300–310 mOsmol) at a drip rate of 1–2 mL/min. Retinorecipient CeA neurons were identified with an infrared differential interference contrast microscope (BX51W; Olympus, Japan) according to their location, and fluorescence illumination was used to excite EYFP fluorescence. The recording pipettes (5–6 MΩ) were prepared with a P97 micropipette puller (Sutter Instrument, USA). In the experiments, patch pipettes were filled with K^+^ solution consisting of 130 mM K-gluconate, 5 mM KCl, 10 mM phosphocreatine, 0.5 mM EGTA, 0.3 mM Na_3_GTP, 4 mM Mg ATP, and 10 mM HEPES (pH 7.2–7.3 adjusted by KOH, 290–300 mOsmol). The resting membrane potentials of neurons were obtained at 0 pA under current clamp. To identify the intrinsic firing pattern of the neuron, we applied a long (1 s) depolarizing current with an initial value of –10 pA in steps of 10 pA while perfusing with CNQX (10 μM) and gabazine (20 μM) to assess retinorecipient CeA neuron excitability without the influence of synaptic currents. ChR2-evoked spikes were then elicited by delivering a 473 nm light stimulus (M470L5, Thorlabs) with 5 mW intensity, 5 ms pulse width, and a frequency of 3–10 Hz while maintaining a 2-s subthreshold current injection from the membrane potential of –70 mV. The Event Detection function in PATCHMASTER software was used to quantify the number and amplitude of the spikes. All recordings were obtained using a HEKA amplifier with a low-pass filter set at 3 kHz and digitization at 10 kHz (HEKA double EPC-10). The series resistance (R_s_) and input resistance (R_in_) were measured at a voltage input of 0.5 mV (V_s_) with a 40-ms-wide depolarization. If R_s_ changed by > 20% before and after recordings, the data were excluded. The acquired data were analyzed offline using PATCHMASTER software (HEKA) and the Mini Analysis program (Synaptosoft version 6.0.3). Raw patch-clamp data were processed using the Savitzky–Golay convolution smoothing method in Origin 2024 software.

### Statistical analysis

During data processing and analysis, the analysts were blinded to the experimental interventions. All statistical analyses were performed using GraphPad Prism 9.0 (GraphPad Software, USA). All data were expressed as mean ± SEM. Data were analyzed using one- or two-way ANOVA with Sidak’s multiple comparisons test or Student’s *t*-test, where appropriate. *P* < 0.05 was considered statistically significant. *P < 0.05; **P < 0.01; ***P < 0.001; ****P < 0.0001; ns, not significant.

## Supplementary information

Supplementary information, Figure S1

Supplementary information, Figure S2

Supplementary information, Figure S3

Supplementary information, Figure S4
