# Supplementary material for: 40 Hz light flickering alleviates chronic pain via adenosine signaling in the retina-amygdala pathway
Source: Cell Res. 2026 Mar 4;36(6):440–61. doi: 10.1038/s41422-026-01227-7 (PMC13201567; doi:10.1038/s41422-026-01227-7)
Supplement: Supplementary file 1 — Supplementary information, Figure S1 [file 41422_2026_1227_MOESM1_ESM.pdf]

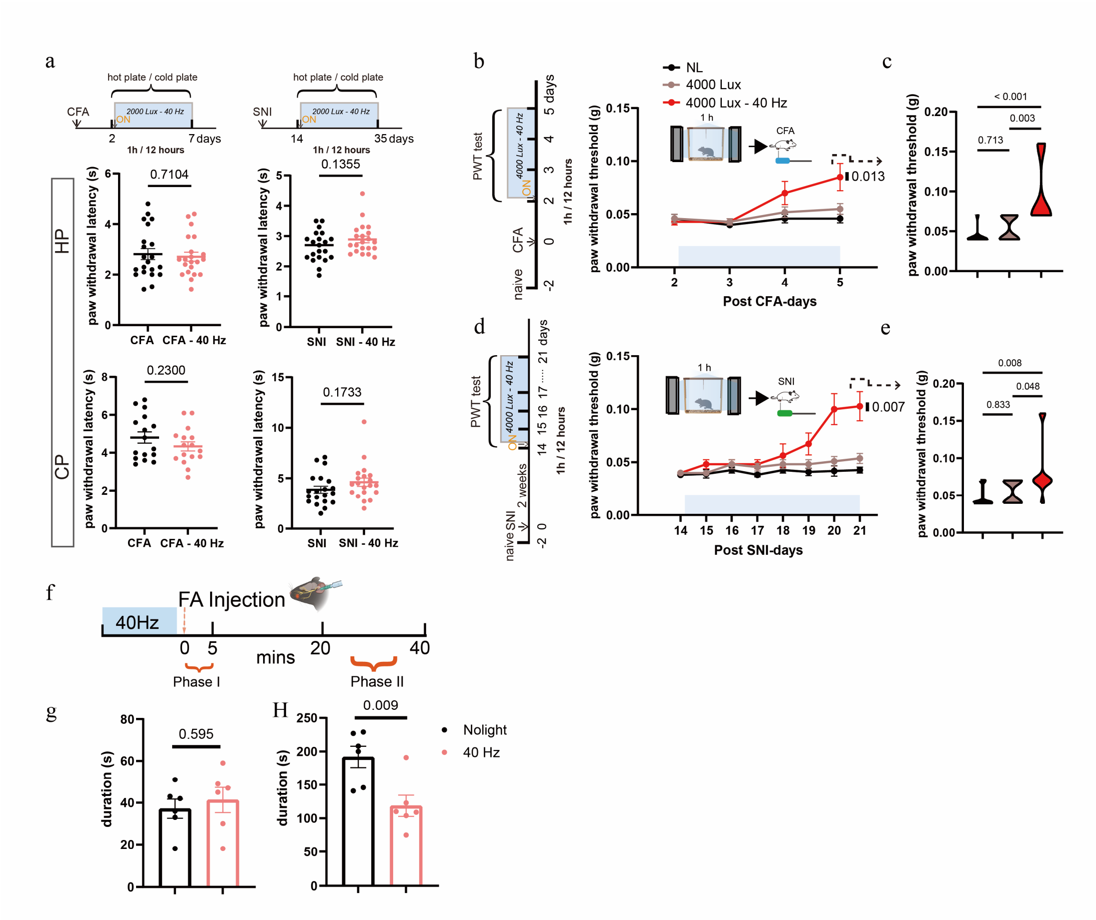

Fig. S1 40 Hz light flickering at 2000 lux significantly reduced spontaneous pain, but did not change thermo sensitivity in both CFA and SNI models, and 40 Hz light flickering at 4,000 lux effectively attenuated mechanical hypersensitivity in both CFA and SNI models.

a The 2000 lux 40 Hz flickering light did not significantly alter responses to either cold plate (4°C, CP) or hot plate (55°C, HP) in both models.

b *Left:* Schematic of the experimental design for 40 Hz light stimulation at 4000 lux and direct current (DC) at 4000 lux in the CFA mice.

Right: PWTs in CFA model mice before and after 2 hours of daily light stimulation. PWTs at various time points during light stimulation. It took three days of 40 Hz light flickering at 4000 lux to significantly reduce mechanical hyperalgesia, as shown by increased PWTs on CFA day 5 compared to the baseline (CFA day 2). Meanwhile, DC at 4,000 lux had minimal effects on PWTs in CFA model mice. Error bars represent SEM. Numerical labels indicate p-values from within-group. For behavioral tests, n = 10 mice in each of the NL (no light stimulation), DC at 4000 lux, and 40 Hz light flickering at 4000 lux groups.

c Summary of results in **b**. Error bars represent SEM. Numerical labels indicate p-values from between-group comparisons.

d *Left:* Schematic of the experimental design for 40 Hz light stimulation at 4000 lux and direct current (DC) at 4000 lux in the SNI mice.

Right: PWTs in SNI model mice before and after 2 hours of daily light stimulation. PWTs at various time points. It required seven days of 40 Hz light flickering at 4000 lux to significantly attenuate mechanical hyperalgesia, with PWTs on SNI day 21 elevated relative to the baseline

(SNI day 14). Meanwhile, DC at 4000 lux had minimal effects on PWTs in SNI model mice. Error bars represent SEM. Numerical labels indicate p-values from within-group comparisons. For behavioral tests, n = 11 mice in each of the NL (no light stimulation), DC at 4000 lux, and 40 Hz light flickering at 4000 lux groups.

e Summary of results in **d**. Error bars represent SEM. Numerical labels indicate p-values from between-group comparisons.

f Schematic of the experimental design for formalin-induced spontaneous pain testing, FA-formalin.

g The 2000 lux 40 Hz flickering light significantly reduced Phase II scratching behavior in formalin-induced spontaneous pain.
