# Supplementary material for: 40 Hz light flickering alleviates chronic pain via adenosine signaling in the retina-amygdala pathway
Source: Cell Res. 2026 Mar 4;36(6):440–61. doi: 10.1038/s41422-026-01227-7 (PMC13201567; doi:10.1038/s41422-026-01227-7)
Supplement: Supplementary file 2 — Supplementary information, Figure S2 [file 41422_2026_1227_MOESM2_ESM.pdf]

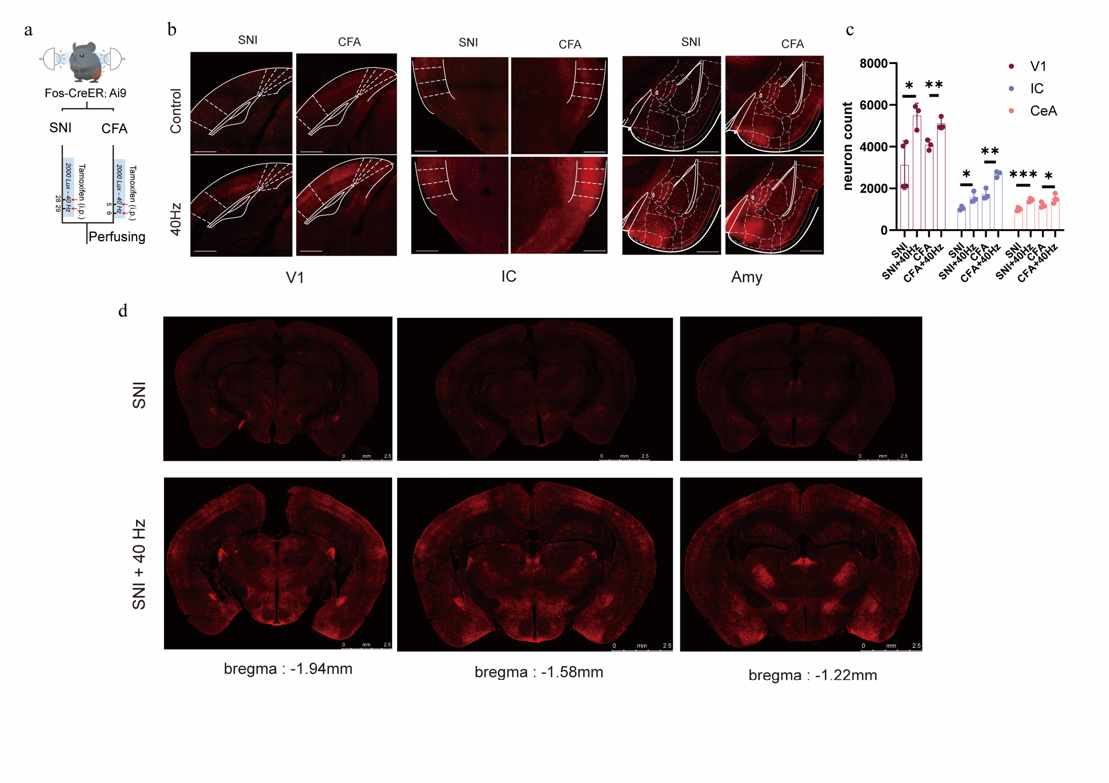

Fig. S2 Overview and quantification of TRAPed neurons in multiple brain regions.

a Schematic illustrating TRAPed neurons activated by 40 Hz light flickering at 2000 lux in both CFA and SNI models.

b Overview of TRAPed neurons in the visual cortex (V1), insular cortex (IC), and amygdala (Amy) in both pain models before and after light stimulation. Scale bar, 500 μm.

c Quantification of TRAPed neurons shown in **b**.

d Representative whole-brain images of TRAPed neurons in SNI mice with or without 40 Hz light treatment. Scale bar, 2.5 mm.
