# Supplementary material for: 40 Hz light flickering alleviates chronic pain via adenosine signaling in the retina-amygdala pathway
Source: Cell Res. 2026 Mar 4;36(6):440–61. doi: 10.1038/s41422-026-01227-7 (PMC13201567; doi:10.1038/s41422-026-01227-7)
Supplement: Supplementary file 3 — Supplementary information, Figure S3 [file 41422_2026_1227_MOESM3_ESM.pdf]

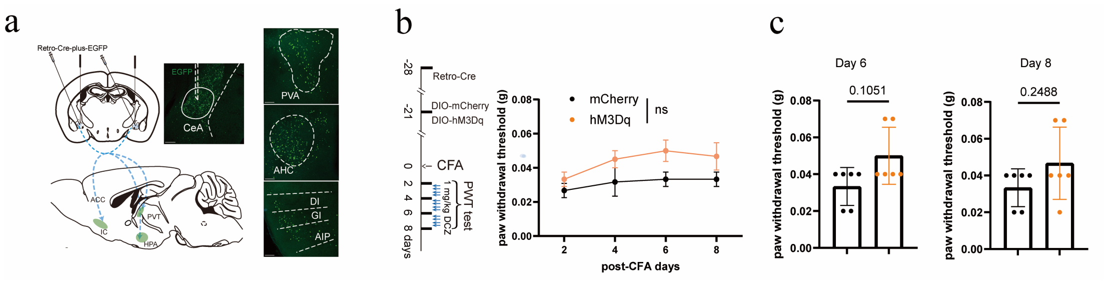

Fig. S3 Chemogenetic activation of CeA-projecting RGCs produced only a modest increase in PWTs in CFA model mice.

a *Left:* Schematic of retrograde labeling of CeA-projecting neurons in the mouse brain.

Right: Representative images showing the retrogradely labeled CeA-projecting neurons in several upstream brain regions. Scale bar, 100 µm.

b Chronic chemogenetic activation of CeA-projecting RGCs (DCZ, 1 mg/kg, twice daily) from CFA day 2 to CFA day 8 caused a modest increase in the PWTs. Error bars represent SEM. For behavioral tests, n = 6 mice in each group, ns, not significant.

c Summary of results in **b**. Error bars represent SEM. Numerical labels indicate *p*-values from between-group comparisons at CFA day 6 and CFA day 8 versus the control.
