# Supplementary material for: 40 Hz light flickering alleviates chronic pain via adenosine signaling in the retina-amygdala pathway
Source: Cell Res. 2026 Mar 4;36(6):440–61. doi: 10.1038/s41422-026-01227-7 (PMC13201567; doi:10.1038/s41422-026-01227-7)
Supplement: Supplementary file 4 — Supplementary information, Figure S4 [file 41422_2026_1227_MOESM4_ESM.pdf]

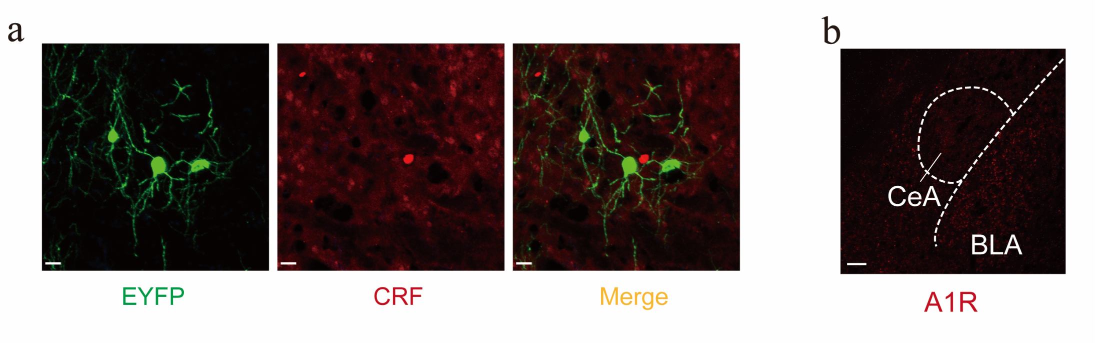

Fig. S4 CRF and A_1R immunostaining in the CeA.

a Representative images showing that retinorecipient CeA neurons (EYFP) largely do not express CRF. Scale bar, 50 μm.

b Representative images showing that A_1R is strongly expressed in the BLA, but weakly in the CeA. Scale bar, 100 μm.
